# Melatonin–Microbiome Two-Sided Interaction in Dysbiosis-Associated Conditions

**DOI:** 10.3390/antiox11112244

**Published:** 2022-11-14

**Authors:** Mara Ioana Iesanu, Carmen Denise Mihaela Zahiu, Ioana-Alexandra Dogaru, Diana Maria Chitimus, Gratiela Gradisteanu Pircalabioru, Suzana Elena Voiculescu, Sebastian Isac, Felicia Galos, Bogdan Pavel, Siobhain M. O’Mahony, Ana-Maria Zagrean

**Affiliations:** 1 Department of Functional Sciences, Carol Davila University of Medicine and Pharmacy, 020021 Bucharest, Romania; 2 Department of Pediatrics, Marie Curie Emergency Children’s Hospital, 041451 Bucharest, Romania; 3 Section Earth, Environmental and Life Sciences, Research Institute of the University of Bucharest, 050663 Bucharest, Romania; 4 Academy of Romanian Scientists, 54 Splaiul Independentei Street, District 5, 050094 Bucharest, Romania; 5 Department of Anesthesiology and Intensive Care I, ‘Fundeni’ Clinical Institute, 022328 Bucharest, Romania; 6 Department of Pediatrics, Carol Davila University of Medicine and Pharmacy, 020021 Bucharest, Romania; 7 Department of Anatomy and Neuroscience, University College Cork, T12 XF62 Cork, Ireland; 8 APC Microbiome Ireland, University College Cork, T12 YT20 Cork, Ireland

**Keywords:** melatonin, antioxidant, circadian rhythm, microbiota–gut–brain axis, dysbiosis, inflammatory bowel disease, chronodisruption, obesity, COVID-19

## Abstract

Melatonin is a pineal indolamine, allegedly known as a circadian rhythm regulator, and an antioxidative and immunomodulatory molecule. In both experimental and clinical trials, melatonin has been shown to have positive effects in various pathologies, as a modulator of important biochemical pathways including inflammation, oxidative stress, cell injury, apoptosis, and energy metabolism. The gut represents one of melatonin’s most abundant extra pineal sources, with a 400-times-higher concentration than the pineal gland. The importance of the gut microbial community—namely, the gut microbiota, in multiple critical functions of the organism— has been extensively studied throughout time, and its imbalance has been associated with a variety of human pathologies. Recent studies highlight a possible gut microbiota-modulating role of melatonin, with possible implications for the treatment of these pathologies. Consequently, melatonin might prove to be a valuable and versatile therapeutic agent, as it is well known to elicit positive functions on the microbiota in many dysbiosis-associated conditions, such as inflammatory bowel disease, chronodisruption-induced dysbiosis, obesity, and neuropsychiatric disorders. This review intends to lay the basis for a deeper comprehension of melatonin, gut microbiota, and host-health subtle interactions.

## 1. Introduction

Melatonin (MT), also known as N-acetyl-5-methoxytryptamine, is conventionally synthetized in the pineal gland through an enzymatic pathway from L-Tryptophan—its first acknowledged function being that of a circadian rhythm regulator. The pattern of its secretion influences a variety of body functions, including temperature, sleep/wake cycle, cortisol secretion, blood pressure, cell proliferation, and immune system activity [1]. MT was first isolated by Lerner in 1958 from a bovine pineal gland extract [2]. Ever since, the published literature agreed on attributing multiple roles to MT. Exogenous MT has also been researched as a potential treatment for a variety of medical and surgical conditions, with positive findings [3,4,5]. Moreover, no studies have indicated any serious adverse effects of MT, making this indolamine a safe and effective therapeutic strategy [6].

MT exerts its activity both through receptor-mediated mechanisms and directly as a redox modulator [7]. Its high lipophilicity enables it to act through direct mechanisms as an oxidative stress radical scavenger, while the presence of MT receptors outside the central nervous system mediates its functions in multiple organs, as previously described in detail by Chitimus et al. [8]. Moreover, MT-synthesizing enzymes have been detected in extra-pineal tissues including the skin, liver, immune system cells, placenta, reproductive tract, and endothelial cells [9,10]. Some authors suggest that MT may be present in the mitochondria of virtually all normal cells [11].

Recent studies described the presence of MT in the cells of the gastrointestinal tract (GIT) [12] as well as in every compartment (lumen, mucosa, and muscularis) and segment, from the esophagus to the rectum of the GIT [13,14,15]. MT functions in the gut are not completely elucidated, but available data support its role in immunity, gastrointestinal (GI) secretion, and intestinal motility [16].

The gut microbiota represents the complex collective community of microorganisms residing in the intestine that coexist with the host in an intricate symbiosis. Intestinal bacteria roles have been extensively studied over the past few years, where their optimal abundance and diversity have been associated with a healthy host. Additionally, various pathologies are linked to gut microbiota disruption, known as dysbiosis [17,18].

Major interactions have been identified between the gut microbiota and melatonin, where enteric communities contribute to the biotransformation and metabolism of tryptophan to serotonin and eventually to melatonin [19], while this indolamine has been shown to have a beneficial effect on intestinal barrier function and microbial communities [20,21].

The rising incidence of dysbiosis-associated pathologies [22] increases a need for new safe and effective therapeutic strategies. In this context, the melatonin–microbiome relationship may be harnessed to develop a novel therapeutic approach. This review has two aims: first, to summarize the melatonin–microbiome interplay and cumulative modulatory effects on the intestinal barrier integrity and function, and second, to support MT as a versatile therapeutic tool in gut dysbiosis-linked conditions.

## 2. Melatonin in the Gut

The idea of extra-pineal MT production was considered after the melatonin-synthesizing enzymes arylalkylamine-N-acetyltransferase (AANAT) and ASMT were discovered in the gut [23,24]. Using immunohistochemistry and radioimmunoassay techniques, Bubenik and collaborators mapped the distribution of MT throughout the rat GIT, identifying higher levels in the duodenum, which tended to decline through the remaining small intestine before rising again toward the rectum [13]. However, a couple of years later, the same author identified different MT levels in the rat GIT, with the peak tissue concentration in the ileum and jejunum, followed by the colon and stomach [25]. Despite the apparent inconsistency within these studies, MT presence in the gut is undisputed. Moreover, MT and its binding sites were also found in the GIT of prenatal and postnatal vertebrates ranging from fish to birds, as well as a variety of mammalian species, including humans [13,15,25,26,27,28,29,30].

The GIT is a major source of MT synthesis independent of pineal production. According to rodent studies, pinealectomy does not influence the intestinal MT concentrations [25]. At least 400 times more MT is present in the gut than in the pineal gland, and 10 to 100 times more than in plasma [28]. In pinealectomized rats, the administration of L-tryptophan (Trp), melatonin’s precursor, enhanced serum levels of MT, strongly supporting MT synthesis in the GIT [31]. In contrast to the well-established pattern of secretion in the pineal gland, no photoperiodic cyclical secretion of MT has been observed in the gut [16], but was rather correlated to the periodicity of food intake [12].

### 2.1. Synthesis in the GIT

Since both the enzymes responsible for MT’s synthesis and immunoreactivity for MT were found in the serotonin-rich enterochromaffin cells (ECs) of Lieberkuhn’s crypts, they are responsible for the intestinal production described below [12,32,33].

In the gut, MT synthesis includes a pathway similar to its synthesis in the pineal gland, requiring the presence of its necessary precursor Trp. This molecule is an essential amino acid, supplied only through the dietary intake of certain foods such as white meat and dairy derivatives [34].

**Figure 1:**
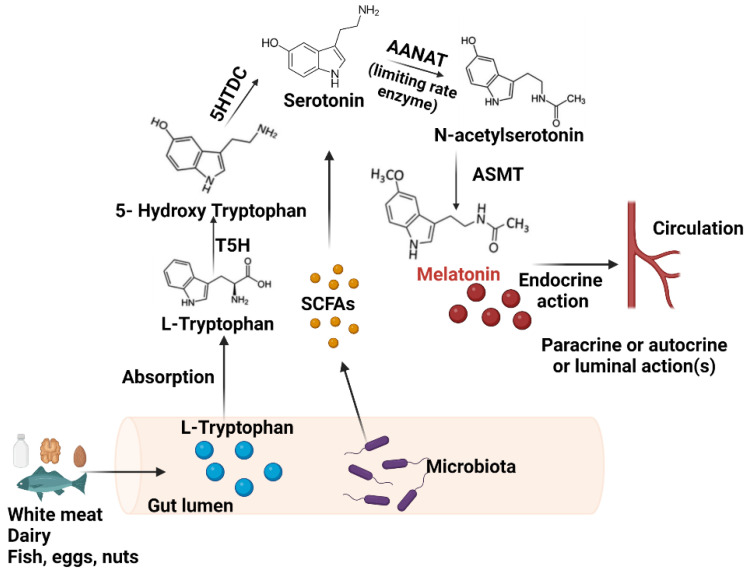
Melatonin biosynthesis in the enterochromaffin cells in the gut (arrows indicate the direction of the reactions). The synthesis starts with L-Tryptophan (Trp), which is first hydroxylated to 5-hydroxytryptophan, followed by decarboxylation to 5-hydroxytryptamine (5-HT/serotonin). The rate-limiting enzyme AANAT converts 5-HT into N-acetyl 5-HT in the following step, and then the ASMT enzyme finishes the synthesis with MT production. After synthesis, melatonin is released in the bloodstream or the gut lumen and exerts local and systemic effects. Tryptophan-enriched diets (e.g., white meat, dairy) or melatonin-enriched diets (e.g., fish, eggs, nuts) can increase the gut production and concentration of melatonin. SCFAs can stimulate the enterochromaffin cells to synthesize serotonin, thus, increasing melatonin’s abundance in the gut. Abbreviations: T5H, tryptophan 5-hydroxylase; 5HTDC, 5-hydroxy-tryptophan decarboxylase; AANAT, arylalkylamine-N-acetyl transferase; ASMT, N-acetylserotonin O-methyltransferase; SCFAs, short-chain fatty acids.

Trp is first converted to 5-hydroxytryptophan (5-HTP), which is then decarboxylated into 5-hydroxytryptamine (5-HT/serotonin) by an enzyme called aromatic amino acid decarboxylase. Trp is used in the human gut to produce 95% of the body’s serotonin, which regulates intestinal motility, gut absorption and secretion processes, and local vasomotricity, and is not able to cross the blood–brain barrier [35,36]. The limiting-rate enzyme AANAT then converts 5-HT into N-acetyl 5-HT, and ultimately, the ASMT enzyme produces MT, which is then released into the bloodstream (Figure 1) [19]. Besides the conversion to MT, Trp undergoes two additional metabolic pathways in the gut. The kynurenine pathway, the way for removing excess Trp, has end-products of kynurenic acid, an NMDA-receptor antagonist; and quinolinic acid, an NMDA-receptor agonist and precursor for NAD-niacin antioxidants [37,38]. The other pathway includes the direct degradation of Trp by colonic bacteria to numerous indole derivatives, including indole, indole-3-propionic acid (IPA), and so on [39]. The increased Trp metabolism on any of the other two pathways in the gut or a low-Trp diet reduces the availability of this molecule for 5-HT, and thus, for melatonin synthesis.

Intestinal MT concentrations depend on food intake, peaking at 2–3 h after eating, and on dietary composition, with rich-Trp diets enhancing the MT synthesis independent of the light–dark cycle [19]. Of note, the consumption of foods containing MT (e.g., fish, eggs, nuts) can lead to considerable increases in the serum level of this indolamine [40].

### 2.2. Receptor Expression in the Gut

The GIT is the largest source of enzymes for MT production, also displaying a high expression of its receptors [25]. Some of its physiological effects are mediated by the activation of specific membrane receptors, classified as MT1, MT2, and MT3, present throughout the GIT, from the esophagus to the colon [16].

MT1 and MT2 receptors belong to the G-protein-coupled receptor family and share a seven-transmembrane structure. However, they activate completely different intracellular signaling pathways [41]. The G proteins that mediate the inhibition of adenylate cyclase and the activation of phospholipase C beta are connected to the MT1 receptor. On the other hand, MT2 interacts with the synthesis of phosphoinositol, inhibition of adenylate cyclase, and inhibition of the soluble guanylate cyclase pathways [42,43,44]. MT3 is an enzyme named quinone reductase 2, that may be responsible for the protective effects of melatonin against oxidative stress [45]. Besides these membrane receptors, MT can bind in the GIT to several nuclear receptors such as the superfamily retinoid Z receptor (RZR)/retinoid orphan receptor γ (RORγ) to display immunomodulatory and anti-cancer effects [46,47].

MT effects in the gut are involved in regulating the immune system, GI secretion, intestinal motility, and the release of peptides involved in energy balance, such as peptide YY [16,48]. MT from the gut is released into the bloodstream or can reach the smooth muscles’ outer layer through diffusion, causing muscle relaxation by counteracting serotonin’s action [31]. In addition to circulation, MT is delivered into the gut lumen to stimulate the postprandial secretion of pancreatic enzymes [49]. MT also controls the mitotic activity, the water content in the gut, and the transmembrane transfer of ions and electrolytes [19]. Moreover, MT produced by the proximal duodenal ECs following neural stimulation binds to MT2 receptors and leads to the release of calcium and bicarbonate ions from nearby cells. Thus, as MT is discharged into the duodenum, the stomach’s acid content is neutralized [50]. The evidence for the numerous roles of MT in the GIT support further research to clarify the complex mechanisms through which MT influences the gut.

## 3. The Gut Microbiota

The human gut microbiota is a complex community of bacteria, archaea, and unicellular eukaryotes [51], which acts as an adaptive interface with the environment. It represents a dynamic entity, whose diversity and composition change throughout human development. The GIT is colonized immediately after birth and, during the first 1000 days of life, the microbiota undergoes substantial and dynamic changes [52]. Moreover, it can be influenced by numerous factors [53], such as diet, antibiotic use, and stress, which significantly affect the health of individuals [54,55,56].

*Firmicutes* and *Bacteroidetes*, together making up more than 90% of the entire bacterial community, are the two dominating phyla in the human gut [57]. Other subdominant phyla include *Proteobacteria, Actinobacteria*, and *Verrucomicrobia* [58]. Alterations to the *Firmicutes* to *Bacteroidetes* ratio have been linked to dysbiosis and several clinical diseases [59]. Additionally, the *Proteobacteria* phylum, present in low amounts in the healthy human gut, is considered the hallmark of dysbiosis when it is increased [60]. The amount of bacterial species in the gut microbiome is called richness and its diversity refers to the number of individual bacteria from each bacterial species that reside there [61]. These terms, the richness and the diversity of the gut microbiota, are two crucial principles that deserve special attention.

The gut microbiota is regarded as a metabolic organ due to the capability of its bacterial communities. They can provide defensive, immunologic, and metabolic activities as a result of active mutualistic interactions between microorganisms and the host. The main metabolites produced by the microbiota are short-chain fatty acids (SCFAs)—namely, butyrate, propionate, and acetate—which are produced in the colon by the anaerobic microbial fermentation of starch and dietary fibers [62]. Through a variety of local effects, including the preservation of intestinal barrier integrity, mucus formation, and anti-inflammatory defense, SCFAs are pivotal for maintaining gut health [63]. Additionally, they also display systemic properties, modulating the immune function through the differentiation of regulatory T cells (Tregs) and anti-inflammatory effects [64,65,66], maintaining energy homeostasis and metabolism, and inhibiting tumor cell proliferation [62,66]. The composition of the gut microbiota also impacts the balance between the intestinal synthesis of antioxidants and the production of reactive oxygen species (ROS) [67].

It is now well known that enteric bacteria exert effects beyond the boundaries of the GIT. Hence, changes in the composition of the gut microbiome can affect the health of the host. For example, inflammatory bowel diseases (IBDs) are chronic disorders characterized by the recurrent inflammation of the GIT. Observations gathered from preclinical and clinical studies also support the link between gut microbiota and IBDs [68]. Several metagenomic analyses of fecal bacteria revealed the presence of significant dysbiosis in IBD patients [69]. IBD-related dysbiosis is characterized by decreased SCFA production, an increase in proinflammatory bacteria [70], and also a reduced diversity of commensal bacteria with increased ROS production [67]. Although it is unknown if this dysbiosis is a cause or a symptom of IBD, the use of microbiome-targeted therapies has shown promising results [71]. Moreover, disturbances in the intestinal microenvironment are frequently associated with prevalent metabolic diseases, such as obesity, type 2 diabetes, and metabolic syndrome, which can lead to atherothrombosis [63].

A wealth of research has suggested that disruptions of the intestinal milieu can be associated with many neuropsychiatric diseases such as autism spectrum disorders, depression, and dementia [72]. Thus, the gut microbiota plays a pivotal role in the proper brain function and behavior via the so-called microbiota–gut–brain axis [18]. This is especially important for microbiome-targeted therapies, particularly in disease entities currently without causal treatment.

## 4. Melatonin and the Gut Microbiome Interplay

### 4.1. The Influence of Gut Bacteria on Melatonin

Melatonin and intestinal bacteria appear to have an intricate functional interrelationship. As discussed below, microorganisms can modulate the synthesis of melatonin in the GIT; conversely, MT is linked to the proper composition and dynamic of the gut microbiota. Together, they form a complex system that acts on multiple levels to maintain the homeostasis of the host.

Various bacteria have an impact on the intestinal synthesis of MT. For instance, a symptomatic *Helicobacter pylori* infection of the gastric mucosa downregulated the expression of the melatonin-producing enzymes (AANAT and ASMT) and reduced the MT production in the GIT. Following the clearing of the infection, MT production returned to normal levels [73].

Beneficial bacteria, known as probiotics, are effective in various dysbiosis-associated disorders, although the precise mechanisms remain unclear. For example, in patients with irritable bowel syndrome, the administration of a multistrain probiotic (VSL#3) improved disease symptoms, exhibiting a positive correlation with the morning systemic levels of MT. The putative underlying mechanism points toward the beneficial role of the probiotic in stimulating MT production [74]. Additionally, the short-term administration of the probiotic *Lactobacillus rhamnosus* can increase the abundance of MT receptor genes in zebrafish, indicating similarity to the consequences of a photoperiod shift to continuous darkness [75]. Thus, probiotics appear to have the potential to influence MT production to alleviate various disease states. Conversely, dysbiosis and subsequent intestinal injury might reduce the local gut and systemic MT levels [76,77].

Enteric populations can influence MT levels via the modulation of its necessary precursors, Trp and 5-HT. The availability of Trp can be modified by dietary changes [78] or by altering the gut microbiota composition, as seen in germ-free mice [79].

Moreover, all three pathways of Trp metabolism in the gut can be modulated by intestinal bacteria. Concerning the indole pathway, the microbiome profile dictates the indole derivative types. These indole derivatives have a wide spectrum of effects, ranging from beneficial outcomes on the intestinal mucosa and immune system to nephrotoxicity. Part of them activates the aryl hydrocarbon receptor (AhR), a nuclear receptor involved in the innate immune response and the maintenance of gut barrier integrity [38,80]. Additionally, Toll-like receptors (TLR) activated by inflammatory stimuli and pathogen-associated molecular pattern (PAMP) molecules released by the gut bacteria stimulate the kynurenine pathway [37].

SCFAs produced in the intestinal lumen stimulate the ECs to release serotonin and enhance MT production [31]. Other metabolites produced by the endogenous spore-forming bacteria can activate the ECs and promote 5-HT biosynthesis in the colon [81]. In a germ-free (GF) mouse model, plasma 5-HT levels were reduced compared to conventional mice. Although gut 5-HT synthesis does not require enzymatic bacterial activity, microbiota indirectly modulates the 5-HT pathway [79]. Furthermore, antibiotic-induced dysbiosis enhances the indole pathway, inhibits the kynurenine route, and decreases colonic 5-HT in animal models [37].

### 4.2. Melatonin’s Role on the Gut Microbiota

**Figure 2:**
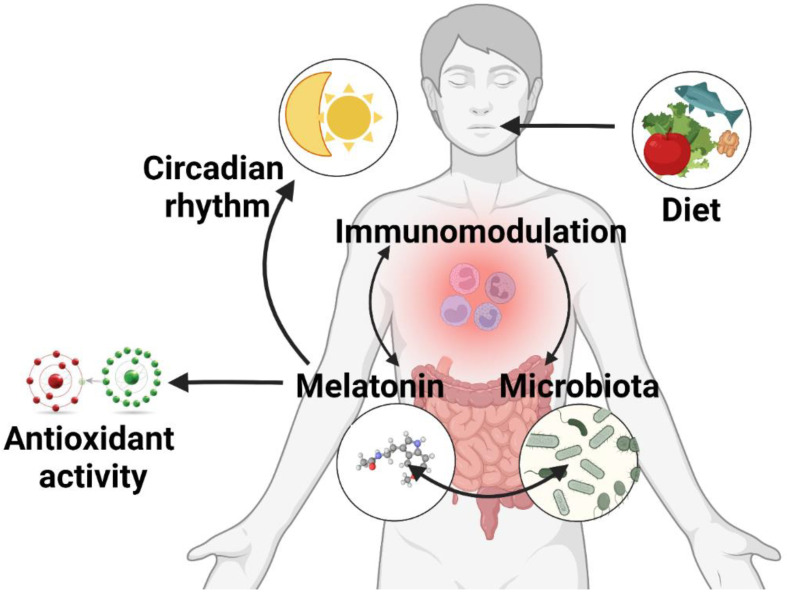
Diagram illustrating the interplay between melatonin and the gut microbiota. Melatonin indirectly modulates the enteric microenvironment through its systemic properties as an immunomodulator, antioxidant, and circadian rhythm regulator. Conversely, the re-established gut microbiota enhances this effect and contributes to the maintenance of body homeostasis. The intestinal bacteria impact melatonin production in the gut by influencing its synthesizing enzymes and precursors. Directly, melatonin could modulate the gut microbiota through molecular mimicry and antibacterial actions on pathogens, but most of the mechanisms warrant further research.

Accumulating evidence shows that MT can modulate the composition and abundance of the gut bacterial population in normal circumstances [82], but especially in various pathological states, as will be detailed below. MT indirectly influences the gut microbiota through its properties as an antioxidant, immunomodulator, and circadian rhythm regulator, but also directly through mechanisms that warrant further research (Figure 2). The latter was demonstrated using GF and antibiotic-treated models that managed to suppress the systemic effects of exogenous MT administration [83,84].

#### 4.2.1. Circadian Rhythm Modulation and the Microbiome

In humans, MT secretion occurs mostly at night in a circadian manner, and maximum plasma levels are registered around 2 to 4 a.m. [85]. The rhythmic release of MT is regulated by the SCN in the anterior hypothalamus, representing the central circadian rhythm generator [86]. The SCN receives optical information from the retina through the retinohypothalamic tract [87], formed by the axons of specialized cells that receive light stimuli without being involved in vision. They represent a subpopulation of ganglion cells, melanopsin-positive, totaling approximately 1–2% of all retinal ganglion cells [88]. Axons of suprachiasmatic neurons project to adjacent hypothalamic nuclei, thalamus, and the brain stem, and synchronize some components of the circadian rhythm, such as the sleep/wake rhythm, feeding schedule, and the activity of the pituitary/adrenal axis [86]. The mechanism of circadian rhythm generation resides in the expression, in all normal cells, of a set of clock-related genes which undergo 24 h cycles of transcription [89]. MT influences the SCN directly by inhibiting neural activity [90], inducing a phase shift [91], and modulating the transcription of clock genes [92]. It was also proposed that MT directly regulates peripheral circadian rhythms [93]. This effect has already been confirmed in some organs and systems, such as the adrenal gland [94], the cardiovascular system [95], and the skin [96].

Circadian rhythms directly or indirectly control numerous metabolic functions through enzyme expression and function [97]. The GIT, and the microbiota, as more recently demonstrated, exhibit variations interconnected with circadian rhythmicity [98,99,100]. In vitro studies showed that various intestinal bacteria are influenced by light–dark cycles and are in close relation with the molecules involved in biorhythmicity. The microbiome diurnal pattern is impaired in the presence of CLOCK mutations, even in the physiological light exposure of the host. On the other hand, if the food consumption timing is regulated in CLOCK-mutant mice, the microbial periodicity is restored [101]. Furthermore, the disruption of the host circadian clock by BMAL1 deletion alters fecal microbial composition [99,102,103]. Dysfunctional circadian rhythms can both cause and exacerbate inflammation in IBDs [100].

Besides the insight concerning the influence of the host’s biorhythm on the microbiota function, studies show that there is, in fact, a crosstalk between the two. The diurnal variation of microbial metabolites, such as butyrate and hydrogen sulfide, can affect the host’s body clock [104]. Given that the production of bacterial metabolites is periodic, there is an implied liaison between the microbial function and the host’s circadian rhythm and metabolism [105]. For once, gut microbiota depletion impairs the rhythmic expression of genes in the GIT [101]. Additional research has strengthened this theory and demonstrated that the temporal distribution of the components of the gut microbiota is impacted by the disturbance of the circadian clock, whether caused by dietary restrictions or phase shifting (such as jet lag) [106,107].

Given that MT acts as a key component of the circadian rhythm and that the gut bacteria also exhibit circadian oscillations influenced by those of the host, we can presume that MT might also modulate this aspect of the microbiota. *Enterobacter aerogenes*, bacteria prevalent in the human gut, have their circadian rhythm and react to MT fluctuations during the day. When MT was present, *E. aerogenes* proliferated more rapidly in a dose-dependent manner. Moreover, the same effect was not observed when exposed to Trp, 5-HT, or N-acetylserotonin, highlighting the importance and sensitivity to MT. Further analysis of this bacterium’s motility patterns revealed an innate circadian rhythm synchronized and boosted by MT. Furthermore, *Escherichia coli* and *Klebsiella pneumoniae*, two additional studied bacterial species, did not share the same sensitivity to MT. This finding is explained by a similarity between certain sequences of *E. aerogenes* and MT receptor genes, which were not identified in relation to these two taxa [102]. Another study revealed that a high-fat diet (HFD) can impair the daily oscillations of the enteric bacteria and that MT can normalize these fluctuations, suggesting that this resynchronization might have a therapeutic significance [108].

#### 4.2.2. Antioxidative Function of Melatonin in the Gut

The antioxidant activity of MT resides in various functions, having a direct role in neutralizing free radicals [109,110,111], and an indirect role by increasing the level of antioxidant enzymes (superoxide-dismutase—SOD, glutathione-peroxidase—GSH-Px, and catalase—CAT) [112,113], stimulating glutathione production [114], and increasing the activity of other antioxidants [115]. On the other hand, a healthy gut microbiota also has a considerable antioxidative role. There is direct involvement of commensal bacteria in metabolizing ROS, as the lactic acid-producing bacteria (e.g., *Lactobacillus* spp.) are equipped with lactate oxidase, NADH oxidase, superoxide dismutase, and pyruvate oxidase—which are enzymes that can remove ROS, thus, reducing oxidative stress [116].

Although it may seem that oxygen species are solely negative by-products, they have physiological functions when maintained within a safe range (e.g., mitigating infections). However, at greater concentrations, they are potentially toxic and cause biomolecular damage, such as the oxidation of proteins, lipids, and DNA, which can lead to several cellular dysfunctions, including cell death [117]. Thus, redox homeostasis is required for proper cellular metabolism and function, and the overproduction of ROS leads to oxidative stress and subsequent inflammation via NF-κB activation [118]. The redox imbalance, either in favor of ROS synthesis or ROS deficit, is closely linked to the pathophysiology of various diseases, ranging from gastrointestinal to neurodegenerative disorders [116,119,120,121].

Colon health is significantly influenced by the intestine’s ability to suppress excessive ROS production. Notably, the composition of the gut microbiota changes depending on the redox balance. In mice exposed to oxidative stress, the gut microbiota experienced an increase in *Bacteroidetes* and a decrease in *Firmicutes, Clostridiales, Ruminococcaceae*, and *Oscillospira* [122]. In a model of aging, the older the mice, the higher the ROS production was, with consequent lower *Clostridiales* and increased *Bacteroidetes* abundances [122]. When gut injury and dysbiosis are present, the antioxidant activity of the GIT can be suppressed. For example, in IBD patients, the reduced diversity of commensal bacteria and changes in the composition of microbiota were associated with increased ROS production and an impaired defense system of the intestinal mucosa [67]. In these situations, MT can exhibit its antioxidant activity and reestablish the redox balance, thus, improving the gut microbiota composition. Similar to other organs, in the gut, MT not only scavenges the highly toxic ROS but also upregulates different antioxidant enzymes (GSH-Px, CAT, SOD) and downregulates pro-oxidative ones [31]. MT can exhibit its activity directly by interacting with ROS, or via membrane and nuclear receptors, which operate as a mediator for its indirect antioxidant action. MT activates several stress-responsive genes (e.g., *AMPK, HIFa, Sirt*) in this pathway, which in turn causes an increase in several antioxidant enzymes [31]. Thus, the reduction in oxidative stress on the gut microbiota through all these MT-mediated pathways has a significant effect on the enteric microenvironment.

#### 4.2.3. Immunomodulatory Function of Melatonin in the Gut

Besides its antioxidant and circadian rhythm-regulating actions, MT plays a complex and multifaceted role as a modulator of the immune system. It is considered an “immune buffer” capable of stimulating the immune response in immunosuppression and physiological conditions while downregulating it during inflammation [123]. These actions are mediated through the membrane and nuclear MT receptors identified in a variety of human and animal immune cells [123].

MT’s key function in preserving the defense system of the body is highlighted by the fact that, in mice deprived of MT either by continuous light exposure or pharmacological inhibition with propranolol, there is a deficient humoral and cellular immune response [124]. Moreover, in healthy or immunosuppressed organisms, MT can upregulate natural killer (NK) cells, monocytes [125], neutrophil chemotactic response [126], B cells, T helper 1 (Th1) cytokines, and downregulate the Th2 response [127]. On the other hand, when inflammation is present, MT inhibits neutrophil infiltration [128], reduces the levels of proinflammatory cytokines (e.g., IL-1β, IL-6, TNF-α) [129], and stimulates the anti-inflammatory ones (e.g., IL-10) [130]. It appears that one of the main pathways which mediate this effect is the inhibition of nuclear factor kappa B (NF-κB)—a transcriptional factor that promotes the expression of numerous inflammatory mediators [131].

MT’s dual role in immunity might be one of the mechanisms which explain its considerable influence on the gut microbiota. Thus, as an activator of the immune response, MT may play a critical role in the defense against pathogens in the gut, including bacteria. For instance, in a model of poly-microbial sepsis, MT enhanced the development of neutrophil extracellular traps (NETs) [132]. Intriguingly, MT also possesses a direct antimicrobial effect on several pathogens, as shown by in vitro studies. Some of the proposed mechanisms involve decreases in intracellular availability of substrates essential for bacterial growth, cell division-related gene expressions, and various enzymes [133]. Therefore, it can be hypothesized that the inhibition of pathogens might benefit the development of commensal bacteria.

In the context of colitis, MT prevents intestinal damage due to its anti-inflammatory properties. Since experimental colitis leads to profound dysbiosis [134,135], it can be presumed that MT, by alleviating inflammation, is capable of re-establishing the homeostasis of the intestinal barrier together with the microbiota.

## 5. Melatonin Involvement in Dysbiosis-Associated Conditions via Microbiome Modulation

### 5.1. Melatonin and the Inflammatory Bowel Disease

In the past, the protective role of MT in colitis was demonstrated by numerous clinical and preclinical studies. This effect could be explained by MT’s anti-inflammatory, antioxidant, and cell survival-promoting properties. However, since dysbiosis plays a major role in the pathogenesis of IBD, MT’s capacity to regulate the microbiota might also contribute to its anti-colitic effect. Accumulating evidence supports this alternative mechanism of action of MT in colitis (Table 1).

Firstly, in animal colitis models, MT increased bacterial richness and diversity in the gut [135,136]. This finding might have a translational impact, as a decrease in these parameters strongly correlates with IBD [140,141].

Regarding the microbial composition, a consistent result of MT administration was the increase in the *Firmicutes* to *Bacteroidetes* ratio [134,135,136]. This variable is regarded as a critical index of intestinal homeostasis, and its reduction is particularly associated with IBD [59]. Other specific changes are related to the increase in beneficial bacterial strains and the decrease in detrimental ones. For instance, MT increased the levels of *Ruminococcaceae* [135] and *Coprococcus* [134]—both SCFA-producing bacteria pertaining to *Firmicutes* and known for their decreased abundance in IBD patients [142]. It has also increased the content of *Bifidobacterium* [137], which previously generated positive results as a probiotic in IBD [143], and of *Lactobacillus*, another promising adjuvant therapy in this disease [144]. Conversely, following MT administration, the enteric communities exhibited a significant decrease in *Proteobacteria*, a representative of Gram-negative bacteria (i.e., *Salmonella, E. Coli*, *Campylobacter concisus*) [135], a phylum with an important role in the pathogenesis of IBD [145]. *Streptococcus* spp. may represent another microorganism down-regulated by MT [136] and correlated with disease activity [146]. Another study reported a reduction in *Desulfovibrio*, *Peptococcaceae,* and *Lachnospiraceae* [137]. A clear association was found between *Desulfovibrio* and ulcerative colitis (UC) [147]. *Peptococcaceae*, although less investigated in IBD, were correlated, in some studies, with gut-related inflammation [148,149]. Finally, the exact roles of *Lachnospiraceae* in diseases of the GIT are not specified yet [150], but a link has been found between the abundance of this family of bacteria and stress-induced microbiota change, with a possible impact on IBD pathogenesis [151]. Changes in microbiota composition induced by MT administration in colitis models are summarized in Table 2.

Importantly, all these bacterial composition changes were accompanied by visible clinical enhancement—quantified by numerous histological methods and by the assessment of symptomatology. This may suggest a mediation of MT’s effect by the microbiota modulation, at least to a certain extent. This hypothesis is supported by a study showing that fecal microbiota transplantation (FMT) from MT-treated animals to the untreated group replicates MT’s beneficial effects in colitis. Furthermore, MT’s protective effect disappeared after co-housing [137].

A recent study reveals a possible mechanism through which MT might modulate gut microbiota composition in colitis [135]. This mechanism involves the activation of TLR4—a receptor belonging to the initial line of infection defense, which also possesses a cytoprotective role in colitis through the recognition of commensal bacteria. Thus, it appears that MT improves clinical and histopathological traits in DSS-induced colitis in WT, but not in TLR4 KO mice. Moreover, MT1 receptor expression was increased by MT treatment and displayed a reduced trend in TLR4 KO compared to WT mice, which supports the connection between TLR4 and MT signaling. MT-treated WT mice also displayed specific changes in microbiota with a putative beneficial impact in IBD. In addition, they exhibited an increased number of goblet cells and an enhanced production of antimicrobial peptides (AMPs). Goblet cells exert an anti-colitic effect through the production of mucin, which prevents the contact between the intestinal epithelium and pathogens [152]. The reduction in goblet cell numbers is associated with dysbiosis, but the exact mechanisms are not defined yet [153]. It is of note that mucin is also an important nutritional source for some bacteria [154]. AMPs (which can also be produced by goblet cells) are responsible for suppressing the growth of Gram-negative bacteria that might be involved in IBD pathogenesis. To conclude, MT improves the first line of defense against pathogens and might modulate the availability of bacterial nutrients via a TLR4-dependent signaling pathway.

In contrast, another study reported MT’s positive outcomes in TNBS (trinitrobenzene sulfonic acid)—induced colitis in mice are mediated by the inhibition of inflammation through the down-regulation of the TLR4/Myeloid differentiation primary response 88 (MyD88)/NF-κB pathway [154]. Moreover, TLR4 is overexpressed in IBD, and its activation is linked to intestinal inflammation and ulceration [155]. Conversely, the inhibition of TLR4 signaling by various compounds was associated with improved histological features, with reduced inflammatory cells infiltration, lymphocyte infiltration of lamina propria, mucosal erosion, congestion, edema, and crypt damage in colonic tissue [156,157]. While there is a clear need for further research to elucidate the exact mechanisms, constitutive levels of TLR4 may be needed so that MT can exert its effects. The excess inflammation encountered in this pathology might be subject to MT’s well-known anti-inflammatory properties, hence, the contradictory results regarding TLR4 modulation.

Eventually, as far as the gut microbiota is concerned, MT does not only possess the capacity to modify the bacterial species composition, but it is also able to decrease intestinal bacterial translocation [158]. Indeed, in a colitis model, MT induced the upregulation of tight junction proteins, zonula occludens-1 (ZO-1), and occludin [137], which are key components of the intestinal barrier [159]. Translocation represents a detrimental process that can lead to endotoxemia, which positively correlates with disease activity in IBD [160].

The efficacy of MT as a therapeutic option in IBD is supported by mounting clinical evidence.

A clear benefit of MT in UC patients was demonstrated by the randomized controlled trial (RCT) conducted by Chojnacki et al. [161]. In this study, patients who received MT in addition to mesalazine maintained clinical remission for 12 months, together with normal CRP and hemoglobin levels, as opposed to those who received mesalazine and the placebo. Moreover, in the non-MT group, The Mayo Clinic Disease Activity Index values were significantly higher throughout the study. In another RCT, MT administration for 3 months was effective in decreasing disease activity and improving role-emotional, energy, and general health components on the 36-item short-form health survey (SF-36) quality of life questionnaire; additionally, it decreased fecal calprotectin as a marker of intestinal inflammation [162]. Although MT in Crohn’s disease (CD) was investigated less, a study on both UC and CD patients showed that a 30-day treatment course with MT as an adjuvant therapy markedly improved histological and ultrastructural abnormalities and inflammation as compared to conventional therapy only, in both conditions [138].

Given these results, the proliferation of EC cells and the surge in enzymes involved in MT synthesis at the colonic level of UC patients [139,163] might represent an important adaptive mechanism. In addition, there was a negative correlation between disease severity and MT concentration in the colon [137], also expressed as the urinary excretion of 6-sulfatoxymelatonin (a metabolite of MT) due to its direct association with the number of EC cells [163]. This negative correlation may reinforce the protective role of this compound, with detrimental consequences when its depletion occurs, and could also express the reduced capacity of MT synthesis by the disrupted mucosa.

To sum up, existing studies show that MT is a potential therapeutic agent in IBD. Additionally, recent preclinical studies highlight gut microbiota modulation as a possible mechanism explaining MT’s anti-colitic effect. Further research is needed to clarify this mechanism together with its relevance for human pathology. To this end, the identification of human gut microbiota changes induced by MT administration in IBD might be of interest.

### 5.2. Melatonin in Sleep Disturbance-Induced Colitis

#### 5.2.1. Sleep Physiology and Gut Microbiome

Modern civilization is increasingly characterized by disruptions of the circadian clock. This hallmark of contemporary lifestyle changes is especially encountered in individuals that engage in chronic shift work or experience the “jet-lag” phenomenon by flying across different time zones. Therefore, inadequate sleep contributes to metabolic diseases such as diabetes and obesity [164], cardiovascular problems [165], neurological and cognitive disorders [166], and can increase susceptibility to infections [167]. Since alterations in the enteric microbiome have also been connected to the same disorders [168,169,170], we anticipate that sleep disturbances may disrupt the gut microbiome and contribute to a disease state.

Previous studies in humans have indicated that partial sleep deprivation (SD) can change the composition of the gut microbiome in as little as 48 h [171], while prolonged periods do not appear to have this effect [172]. However, these studies correlating sleep and gut microbiome rarely involve humans and have a small sample size. Moreover, different microbiome sequencing methods may be causing these conflicting results.

Apart from these controversial findings, a handful of preclinical studies have shown significant changes in the composition and diversity of the gut microbiome in several models of pathologic sleep, such as circadian disruption [84,101] and sleep fragmentation [173,174] and deprivation [76,77,135]. These stark discrepancies between humans and murine models may result from different animal models and various sampling techniques (feces vs. colonic content).

In murine models, sleep disruption induced a sizable disturbance in the microbial populations, with alterations in the diversity and richness of the gut microbiome. At the phylum level, it presented with increased *Firmicutes* to *Bacteroidetes* ratio, one of the most important markers of microbiota balance, and *Proteobacteria,* the bacterial hallmark of dysbiosis [76,77,173,175]. At the genus level, it decreased beneficial *Akkermansia, Bacteroides,* and *Faecalibacterium*, and increased pathogenic *Aeromonas, Helicobacter,* and *Clostridium* [76,84,175]. Members of the *Enterobacter* complex can act as opportunistic pathogens [176], and a decreased abundance of *Lactobacillus* and *Akkermansia* can lead to different pathologies [144,177]. To sum up, these microbial changes highlight an important gut dysbiosis induced by pathologic sleep that could lead to various associated diseases.

By activating the NF-κB pathway in the colon, SD affects gut homeostasis, resulting in the downregulation of mucus production, enterocyte proliferation, tight junction protein expression (claudin-1, occludin, ZO-1), and the of number of goblet cells [76]. In terms of the colon’s antioxidant capacity, SD significantly reduces the levels of antioxidant enzymes (GSH-Px, CAT, and SOD) and total antioxidant capability (T-AOC), while increasing the end-product of lipid peroxidation, malondialdehyde (MDA), enhancing the overall oxidative stress in the gut [76]. Ultimately, as expected, sleep disruption leads to a pro-inflammatory state by upregulating pro-inflammatory cytokine levels (IL-1β, IL-6, IL-17, TNF-α), while lowering the anti-inflammatory markers (IL-5, IL-10, IL-22, IFN-α) [76,77].

Pathologic sleep enhances oxidative stress and the pro-inflammatory state, and modifies metabolic pathways related to intestinal homeostasis, eventually leading to specific gut microbiome alterations. Therefore, sleep disruption is associated with the impairment of the global gut diversity and specific changes in bacterial taxa, although these results vary based on the microbiome analysis metric and pathologic sleep model.

#### 5.2.2. Melatonin in Pathologic Sleep-Induced Dysbiosis

Accumulating evidence highlights that MT could modulate gut properties and the resident microbes in response to stress, such as sleep disturbance. Regarding the levels of plasmatic MT and the impact of sleep, preclinical and clinical studies show inconsistent results. In healthy human subjects, acute periods of SD or delayed sleep onset had no effect or enhanced the plasma levels of MT [178,179,180]. However, none of these studies approach the microbiota changes during SD. This leads us to think that plasma MT might be decreased only when significant intestinal damage is present. Moreover, in a preclinical study, the sleep restriction maintained the serum MT levels, suggesting the pineal gland as the main secretory site. However, although gut dysbiosis was present in this case, no histologic intestinal damage was highlighted [21].

When the interaction between MT, SD, and the gut microbiome is assessed, in preclinical studies where evident intestinal damage is present, plasmatic and intestinal MT levels are reduced [76,77]. These findings summarized in Table 3 suggest a possible connection between MT and SD-induced colitis.

Overall, MT supplementation in sleep disturbance-associated colitis reestablished the gut microbiota balance and the integrity of the intestinal barrier. Thus, MT restored the richness and diversity of the gut microbiota [21,76,77]. At the phylum level, it decreased the *Proteobacteria* and *Firmicutes* to *Bacteroidetes* ratio. At the genus level, MT increased *Akkermansia, Lactobacillus, Bacteroides,* and *Faecalibacterium,* which are known beneficial bacteria that could lower inflammation, and decreased colitogenic *Aeromonas* [76,77]. MT significantly increased the *Akkermansia muciniphila* abundance [21,76,84], a bacterium known for its beneficial roles in the gut (e.g., increased mucin production, barrier integrity preservation) [177,184]. Additionally, by increasing the abundances of *Bacteroides* spp., *Lactobacillus* spp., *Akkermansia* spp., and *Faecalibacterium* spp., MT enhanced the levels of butyrate, one of the gut microbiota’s main beneficial metabolites [20]. MT also reestablished the integrity of the intestinal barrier by increasing the expression of the tight junction proteins (claudin-1, occludin, ZO-1), and caspase recruitment domain-containing protein 9 (CARD9) [76,182], a signaling adaptor known to modulate the activation of the innate immunity [185]. Moreover, MT restored the number of goblet cells, mucus production, and enterocyte proliferation impaired by the SD [76,175]. The number of goblet cells and MUC2 protein were, in particular, associated with MT’s capacity of downregulating the abundance of *Aeromonas,* reinforcing a possible microbiome-mediated effect [175].

By increasing the GSH-Px, SOD, and CAT levels, pivotal antioxidant enzymes involved in scavenging harmful ROS and MT also proved to be effective in reducing the associated oxidative stress [76,77]. As considered above, pathologic sleep also creates an important pro-inflammatory systemic state. MT counteracts this effect by reducing the amount of pro-inflammatory cytokine levels (IL-1β, IL-6, IL-17, TNF-α) while increasing the anti-inflammatory markers (IL-5, IL-10, IFN-α, IL-22) [76,77,181]. As a putative mechanism, similar to IBD, MT downregulates TLR4 in sleep-deprived mice, decreasing the probability of prolonged inflammatory responses [183].

Additionally, SD can activate the hypothalamic–pituitary–adrenal (HPA) axis, which triggers the production of corticosterone (Cort) [76,77]. Therefore, a model of gut dysbiosis was induced in mice using Cort administration [182]. MT proved again to represent an effective way to mitigate these effects. MT supplementation reduced the plasma levels of the stress hormone, acting as a homeostatic regulator of the HPA axis [77]. Moreover, MT reestablished the balance of the gut microbiome following Cort exposure [182], reinforcing its protective role in dysbiosis. This indolamine, via the MT2 receptor, blocked the glucocorticoid receptor synthesis and transport, and the activation of the STAT3/AP-1/NF-κB pathway caused by Cort. This further suppressed the oxidative stress, which mediated the imbalance in the intestinal microbiota and its metabolites [182]. Furthermore, by blocking the TLR4/MyD88/GSK-3β/β-catenin/ROS/NF-κB loop, MT via the MT2-mediated pathway restored MUC2 depletion, ultimately alleviating the induced colitis in mice [183].

To sum up, as a result of SD’s inhibition of MT production, the gut experiences oxidative stress and inflammation, which leads to specific alterations in the gut microbiome. MT plays a crucial role by alleviating mucosal damage and dysbiosis. These promising findings suggest that MT could be used as a probiotic to treat SD-related intestinal impairments and to assist in preserving the balance of the gut microbiota. Additionally, rather than the stress caused by the SD per se, MT suppression may be the main culprit causing intestinal damage and dysbiosis.

**Figure 3:**
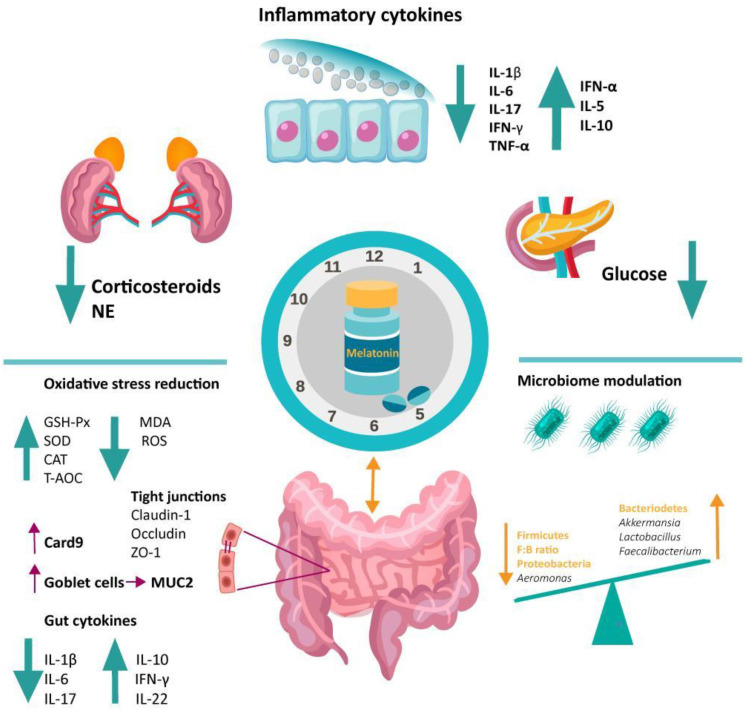
Melatonin’s beneficial systemic and GI effects in sleep disorders. In the gut, melatonin: (i) reestablishes the intestinal microbiome—decreases the *Firmicutes* to *Bacteroidetes* ratio and *Proteobacteria* at the phylum level; increases *Akkermansia, Lactobacillus,* and *Faecalibacterium*; and decreases *Aeromonas* at the genus level; (ii) restores the barrier integrity by increasing tight junction proteins (claudin-1, occludin, ZO-1), goblet cell numbers (MUC2), and CARD9; (iii) reduces the oxidative stress by increasing the levels of the antioxidant enzymes (GSH-Px, SOD, CAT), T-AOC and decreasing MDA, ROS; (iv) diminishes the inflammatory state by decreasing the proinflammatory cytokine (IL-1ß, IL-6, IL-17) and increasing anti-inflammatory ones (IL-10, IFN-γ, IL-22). At the systemic level, melatonin: (i) reduces the stress hormones (corticosterone, NE); (ii) diminishes the inflammatory systemic state by decreasing the inflammatory cytokines (IL-1β, IL-6, IL-17, TNF-α, INF-γ), and increasing the anti-inflammatory ones (IL-5, IL-10, IFN-α); (iii) restores glucose homeostasis by decreasing the glucose levels. Abbreviations: ↑, Increase; ↓, Decrease; ZO-1, Zonula Occludens-1; MUC2, Mucin 2, oligomeric mucus gel-forming; GSH-Px, glutathione peroxidase; SOD, superoxide dismutase; CAT, catalase; T-AOC, total antioxidant capability; MDA, malondialdehyde; ROS, reactive oxygen species; TNF-α, tumor necrosis factor-α; IL, interleukin; IFN- γ, interferon γ; CARD9, Caspase Recruitment Domain Family Member 9; NE, norepinephrine.

In conclusion, the exogenous administration of MT restores the systemic and gut microbiota alterations induced by sleep disruptions (Figure 3), implying a putative role of MT in modulating the microbial communities depending on the health status.

#### 5.2.3. Melatonin, Sleep Disturbance, and IBD

A lack of sleep can harm the gut barrier, including the mucosa, and disturb the intestinal microbiome, increasing the risk of developing IBDs. Additionally, sleep disturbances are typically listed as stressors by individuals with IBD and they are also associated with disease severity [186,187]. In experimental models of IBD, SD was shown to enhance intestinal damage [181,188]. Colitis exacerbated by SD could respond favorably to MT administration. To study its effects, MT was administered to DSS-induced colitis combined with SD in mice. By reducing inflammation and erosion, MT was able to lessen the intestinal damage in addition to preventing weight loss and enhancing survival rates. Furthermore, MT strengthened its well-known anti-inflammatory effects by lowering pro-inflammatory cytokine levels (IL-1β, IL-6, IL-17, TNF-α, and INF-γ) [181].

In IBDs, where gut dysbiosis is strongly postulated, MT has an obvious influence by modulating the intestinal milieu. In sleep disruptions, where the relationship between sleep physiology and bacterial communities is not clearly stated, MT can exert its functions only in specific circumstances when the mucosal impairment and dysbiosis are clearly stated.

### 5.3. Abnormal Light Exposure and Dysbiosis

The alteration of light–dark cycles, a feature of modern society’s lifestyle, impacts not only the neurocognitive development, behavior, feeding habits, offspring development, and reproductive health [189,190,191,192], but also the gut microbiota and body homeostasis. In rodent studies, the disruption of the circadian rhythm altered the intestinal microbiome with a decreased abundance of *Bacteroidetes* and increased *Firmicutes* phyla, with the effects enhanced by a high-fat or -sugar diet [193,194]. Deaver et al. showed that several weeks of continuous light exposure to mice decreased intestinal microbiota diversity and altered its composition. Specifically, chronodisruption increased the abundance of *Ruminococcus torques,* bacteria associated with impaired intestinal barrier integrity; and decreased *Lactobacillus johnsonii*, which regulates carbohydrates, the body’s metabolism, and maintains gut barrier integrity, preventing abnormal inflammation and carcinogenesis [195]. The alteration of the circadian rhythm in rats impaired glucose metabolism and induced reproductive changes similar to polycystic ovary syndrome—effects that were linked to gut dysbiosis [196]. Further, in a study on zebra finches, nocturnal light exposure decreased the abundance of *Lactobacillus* spp. and increased *Proteobacteria* phylum compared to normal light–dark cycle exposure; changes that were associated with body mass gain and fat accumulation in the liver were ameliorated by oral supplementation with *Lactobacillus rhamnosus* [197].

Taken together, all these studies demonstrate that constant light exposure induces dysbiosis and alters gut metabolic products, with a potential impact on the entire body’s homeostasis. Various metabolites of commensal bacteria rhythmically produced, such as SCFAs, depend on gut microbial composition and modulate host metabolic homeostasis by maintaining gut barrier function and by the widespread reprogramming of circadian transcriptional activity [107]. Additionally, it is well known that MT secretion is regulated by light/dark exposure, and darkness stimulates the secretion of MT. A possible pathophysiologic and therapeutic role of MT in the chronodisruption models of dysbiosis and metabolic diseases should be considered [106].

### 5.4. Melatonin and Gut Microbiome Modulation in Other Diseases

#### 5.4.1. Melatonin–Microbiome–Gut–Brain Axis

MT mitigates the effects on multiple neurological disorders [198] and recent studies highlight this hormone’s impact on gut bacteria, suggesting possible crosstalk between MT and the microbiome–gut–brain axis. Thus, the enteric microbiota may play an important role in brain-related diseases also via MT modulation in the gut.

For example, in a zebrafish model, neural hyperactivity was induced by caffeine administration to explore the gut microbiome-mediated regulatory effect of MT and probiotics (*Lactobacillus plantarum*) on neurotransmitter secretion. Caffeine induced imbalances in the brain neurotransmitter concentrations, such as increased dopamine, and reduced GABA and serotonin, and also perturbed the intestinal microbiota. After 14 days of treatment, MT managed to have a better effect than the probiotic and restored the neurotransmitter concentrations to the levels of the control group. Moreover, MT recovered the microbial community structure and restored the SCFA’s metabolism, which further modulated the neurotransmitters. In the validation experiment using germ-free animals, the neurotransmitter recovery and the SCFA content did not reach the same level as in the holobiotic zebrafish after adding the same amount of MT. These results support the potential role of MT through the microbiome–gut–brain axis modulation [83].

Another preclinical study revealed that MT might counteract some of the pathophysiological processes seen in Alzheimer’s disease (AD), with the direct involvement of gut microbiota [199]. The experimental model comprised genetically modified mice lacking the gene for arylalkylamine N-acetyltransferase (*Aanat*)—an essential enzyme in MT synthesis. The first observation was that *Aanat^−/−^* mice exhibited multiple abnormalities of metabolism and gene expression, including genes with a crucial role in neuronal function. They also displayed excessive inflammation and a dysbiotic status, expressed by a decrease in *Bacteroidetes* and a change in the *Firmicutes* to *Bacteroidetes* ratio. However, the rise in *Lactobacillus* was inconsistent with previous studies where MT augmented the levels of this bacteria [136]. These changes were completed with increased values of gut permeability and fecal gut inflammation markers, indicating an affliction of the colonic mucosa. Simultaneously, an AD-like phenotype was observed, with decreased uric acid and increased total tau (T-tau), microglial activation, pro-inflammatory cytokines, and Amyloid beta (Aβ) protein deposition in the brain, together with impaired spatial learning. The possible involvement of the microbiota in these modifications might be inferred from the correlation between increased *Lactobacillus* and decreased uric acid levels. After FMT was performed from WT to *Aanat^−/−^* mice, there was a notable enhancement in gut permeability, systemic inflammation, microglial activation, and Aβ deposition. To conclude, these results reflect MT’s vital role in maintaining the homeostasis of various systems and organs, including the brain, with a remarkable contribution to the microbiota.

Another dysbiosis-associated condition that could be improved by MT administration is represented by spinal cord injury (SCI), as suggested by Jing et al. [200]. Indeed, besides increased gut permeability, inflammation, and GIT motility alteration, it was reported that mice in the SCI group suffered important changes in the composition of the gut bacterial population. Thus, an elevation in *Clostridiales* was observed, together with a decrease in *Lactobacillales* (e.g., *Lactobacillus*, a notable exponent of this order) and in *Bifidobacteriales*. The post-SCI MT intervention managed to reverse these changes (except for the decrease in *Bifidobacteriales*), bringing the overall bacterial population structure closer to that of mice from the sham group. Additionally, MT attenuated motility, colonic inflammation, and the intestinal barrier function and, more importantly, enhanced locomotion (assessed by Basso Mouse Scale and DigiGait). *Lactobacillales* and *Lactobacillus* levels were positively correlated with locomotion scores and negatively correlated with gut permeability, whereas the opposite associations were identified regarding *Clostridiales*. Since these bacteria were influenced by treatment with MT, it was suggested that their variation partly contributed to the protective effect of MT. This possibility was further investigated by the administration of antibiotics before SCI, to deplete the gut microbiota. Subsequently, the beneficial outcomes of MT administration concerning locomotion and intestinal permeability partially diminished, assuming they were mediated to a certain degree by the microbiota. However, MT also enhanced the same functions in antibiotics + SCI + MT mice compared to SCI mice who received antibiotics but not MT, underlining this compound’s capacity of re-balancing the enteric communities.

Collectively, these results support the neuroprotective role of MT concerning microbiota modulation and may represent a foundation for future studies investigating the role of this hormone in neurological conditions with a clear involvement of dysbiosis.

#### 5.4.2. Melatonin–Microbiome in Obesity

Recently, the prevalence of obesity has suffered a dramatic increase, with nearly a third of the total population being categorized as obese or overweight [201]. Given the serious cardiovascular risk and the numerous comorbidities associated with this condition, new ways to address this public health issue are needed.

It is a well-known fact that the microbiota of obese patients undergoes important structural changes as compared to that of healthy individuals [202]. Moreover, it was proved that FMT from humans adopting a Western diet even for a single day can engender an imbalance in the intestinal milieu of germ-free mice [54]. Surprisingly, in germ-free rodents, a high-fat Western-type diet did not manage to induce the usual metabolic alterations, highlighting the vital role that intestinal bacteria play in the pathophysiology of obesity [203]. Therefore, we can posit that MT’s recently discovered capacity of regulating the gut microbiota might contribute considerably to its anti-obesity effect observed in clinical studies.

According to recent preclinical studies, MT reverses most metabolic, clinical, morphological, and gut microbial composition changes generated by a high-fat diet (HFD)—the main rodent model used for investigating obesity. Firstly, MT managed to restore the diversity of the microbiota that had decreased as a result of HFD [204]. Another study reported, on the contrary, that it reduced richness and diversity, but it modified the gut microbial structure to resemble that of normal diet mice [205]. Yildirim and colleagues found that MT augmented the bacterial populations in comparison to the control, but it prevented the abrupt bacterial overgrowth caused by HFD [82]. Interestingly, Yin and colleagues found similarities between the cosine curves of daily oscillations of various taxa in HFD mice who received MT and controls, in contrast to HFD-only mice, proving that MT can also restore bacterial rhythmicity [108]. Collectively, these results show that, even if MT sometimes leads to contrasting effects, they are generally opposite to those produced by a high-fat diet.

Regarding specific taxa, MT consistently prevented the increase in the *Firmicutes* to *Bacteroidetes* ratio [108,204,205]. It also stimulated *Verrucomicrobia*, including *Akkermansia*—an effective probiotic in metabolic disorders [205]. Down-regulation was encountered in *Desulfovibrionaceae* (endotoxin-producing bacteria), *Alistipes*, and *Anaerotruncus*, of which are correlated with obesity [205].

In parallel with these outcomes related to the microbiome, MT led to a significant improvement in the metabolic disorder in HFD rodents. MT decreased the total body weight and the proportion of visceral adiposity and promoted the production of brown adipose tissue and thermogenesis [84,108,205]. It also lowered inflammation, lipogenic gene expression, and cholesterol and triglycerides levels [205]. Furthermore, it re-established the normal circadian variations in the expression of clock genes and in serum triglycerides, which were disrupted by HFD [108]. Additionally, it enhanced glucose metabolism by decreasing glycemia and enhancing insulin sensitivity [205]. Concerning the liver, it counteracted steatosis and inhibited the NF-κB pathway, of which is associated with nonalcoholic fatty liver disease (NAFLD) [205].

A particular mechanism by which MT might alleviate lipidic dysmetabolism is related to the inhibition of *Escherichia coli* [84]. To this end, Rong et al. investigated metabolic syndrome-related changes in a model of jet-lag mice who exhibited an increase in body weight, ileal lipid uptake, epididymal fat, and a decrease in angiopoietin-like 4 (ANGPTL4)—a regulator of lipid metabolism. Simultaneously, an increase in *Escherichia coli* and LPS production was observed. MT reversed all these modifications, and, through the downregulation of *E. coli* together with LPS synthesis, it inhibited the LPS/TLR4/STAT3/NFIL3 pathway, leading to a reduction in lipid uptake. However, microbiota depletion through antibiotic administration neutralized MT’s capacity of reducing ileal lipid uptake and epididymal fat, strengthening the contribution of the enteric communities to MT’s actions.

Other bacteria that could participate in MT’s anti-obesity effect are SCFA-producing species [204]. Thus, while HFD lowered fecal acetic and propionic acid, MT restored acetic acid levels, which were correlated with *Bacteroides* and *Alistipes* (also upregulated by MT). Following sodium acetate administration in HFD mice, the total weight and relative weight of adipose tissue decreased, and the expression of genes involved in lipid metabolism normalized, supporting the beneficial actions of SCFA.

A modality of proving the mediation of MT’s effect on obesity by the gut microbiota is FMT. In this regard, the fecal transplant from MT + HFD mice to germ-free mice, followed by HFD, decreased weight and weight gain and produced microbiota composition changes similar to those elicited by MT treatment, when compared to the transplant from HFD mice [204]. Interestingly, the fecal transplant from mice who were on a normal diet and received MT did not exert similar benefits, which implies that MT’s effects might be diet- and health status-dependent [204]. Another surprising finding is that the moment of the day when FMT is performed can influence its outcomes, leading to differences in serum lipid indexes and the amount of adipose tissue, which emphasizes once again the importance of the circadian rhythm of the gut microbiota [108].

The critical role of MT in lipid metabolism is also underlined by the consequences of its absence. Thus, in the previously mentioned study by Zhang et al. [199], *Aanat^−/−^* mice exhibited high glycemia, altered insulin sensitivity, and NAFLD-associated gene expression in the liver. Following HFD, they also displayed increased body and epididymal white tissue weights, hepatic steatosis, and altered glucose metabolism, in contrast with mice who were not arylalkylamine N-acetyltransferase-deficient. Furthermore, FMT from WT mice to *Aanat^−/−^* mice who received HFD managed to attenuate most of these changes.

In summary, recent data show that gut microbiota has a critical role in the development of obesity and metabolic syndrome. Therefore, MT’s capacity to restore the normal composition and function of this complex structure justifies its use as a medication in these particular pathologies.

### 5.5. Melatonin in COVID-19-Associated Dysbiosis

The COVID-19 pandemic is one of the most devastating events in our recent history. The SARS-CoV2 virus induces this disease characterized mainly by lung injury. However, digestive afflictions can be present [206]. The gut alteration is associated with lower mortality but worse respiratory symptoms in these patients [207]. This could be explained by the gut-lung axis’ anti-inflammatory and immunomodulatory effect on the lungs, probably mediated by IL-10 and interferons [208,209,210]. SARS-CoV2 induces gut impairment by microbiota dysfunction, as it has a high abundance of angiotensin-converting enzyme 2 (ACE2) receptors in the intestine. The ACE2 receptor represents a molecular chaperone for B0AT1—a transporter of neutral amino acids, including tryptophan and glutamine, of which have essential roles in the modulation of the immune response, inflammation, and intestinal barrier integrity. Thus, the internalization of ACE2 and B0AT1 after a SARS-CoV2 infection might be one of the mechanisms leading to dysbiosis [211]. The gut microbiota alteration of COVID-19 patients is characterized by a reduction in anti-inflammatory taxa, with a higher abundance of opportunistic pathogens [211]. Importantly, dysbiosis is correlated with disease severity [212]. There are also some case reports describing severe cases of acute hemorrhagic colitis due to the SARS-CoV2 infection associated or not with respiratory symptoms [213,214,215].

MT was introduced as an adjuvant therapy in COVID-19 treatment from the beginning of the pandemic [216,217]. MT was initiated after the observation of children’s mild symptoms that are known for their high MT levels [218]. MT can improve sleep quality in patients, but also has antioxidant, anti-inflammatory, and immunomodulatory effects [8]. MT’s antioxidant effect mediated by the neutrophil myeloperoxidase inhibition through heme production, catalase-like activity, and HOCl scavenging also has a beneficial effect on COVID-19 [219]. The effect of MT on the gut microbiome is another argument for its usage in COVID-19, thus, improving the outcome. Moreover, several RCTs support the potential role of MT as an adjunctive therapy in this disease [220,221,222].

## 6. Concluding Remarks

Originally, MT was considered to be synthesized exclusively in the pineal gland, and the vast majority of studies focused mainly on its antioxidant, immunomodulatory, and circadian rhythm regulation functions. MT can be also used as a medication mostly recommended for sleep disturbances. However, its localization in the gut opened up new avenues for research, since a growing body of evidence emphasizes that MT has potentially beneficial effects on host health through interactions with the gut microbiota.

**Figure 4:**
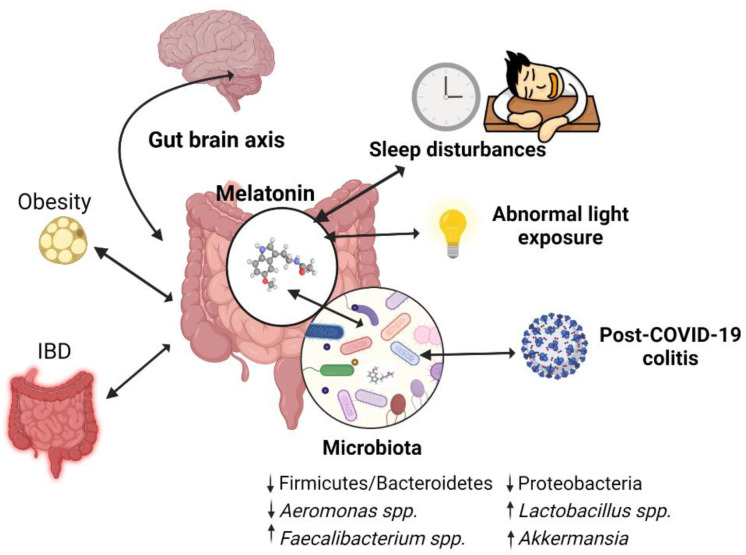
Dysbiosis-associated conditions where melatonin’s effects have been studied. The bidirectional arrows represent the mutual interactions between melatonin and these pathologies: while melatonin alleviates symptomatology through microbiota modulation, the dysbiosis which occurs in these conditions disrupts the intestinal mucosa, leading to a decreased synthesis of melatonin.

Recent scientific evidence reveals that MT and the colonic bacterial populations act synergistically and maintain GI and systemic homeostasis through their multiple actions on immunity, oxidative stress, circadian rhythm, and other functions that remain to be elucidated. These findings may be of particular relevance in the context of the increasing number of studies that show an association between dysbiosis and an array of conditions (e.g., IBDs, chronodisruption-associated dysbiosis, obesity, and neuropsychiatric disorders) (Figure 4), most of them with an unclear pathogenesis still. Although the causal relationship between gut microbiota imbalance and these diseases has not been defined yet, therapeutic agents capable of re-balancing the intestinal milieu hold great promise for improving these conditions. Considering all the gathered studies and presented results, this review hypothesizes that MT might be one of such agents, having the advantage of acting through multiple pathways.

Despite its efficacy, the exact pathways by which MT modulates the microbiota are not known. Whereas immunomodulatory and antioxidant mechanisms (e.g., NF-κB, TLR4, ROS) are likely to play a decisive part, direct effects on intestinal bacteria have not been studied enough. Available data suggest that MT exerts antibacterial actions on pathogens through various mechanisms and could influence certain bacteria due to molecular mimicry. Certainly, the direct impact of MT on gut bacteria is the subject of future mechanistic studies. These are especially justified given the paramount contribution of microbiome-mediated pathways involved in MT’s therapeutic effect and are emphasized by the experimental validation models (e.g., GF and antibiotic-treated animals, FMT). Moreover, the presented data strongly promote further investigations of MT’s probiotic-like effects in conditions with a more recently discovered dysbiotic component, such as cancers, COVID-19, and autoimmune and neuropsychiatric disorders.

In addition, future clinical studies are necessary to determine the efficacy, optimal dose, administration route, and possible limitations. However, it is of note that exogenous MT does not comprise only pharmacological interventions, but also diets containing MT. An even more important source is the MT produced endogenously and modulated through Trp-enriched diets, probiotic administration, a normal sleep/wake cycle, and an eating schedule.

In conclusion, this review offers a new perspective on the functions of MT, with gut microbiota modulation as an alternative mechanism of action. In addition to other properties of this indolamine that have been thoroughly studied in the past, this pathway might explain the clinical efficacy of MT in various pathologies. In its turn, the intestinal milieu seems to have a considerable impact on the availability of MT. Thus, MT and the gut microbiota appear to synchronize and potentiate each other, being part of a two-sided interaction in dysbiosis-associated conditions.

## Figures and Tables

**Table 1:** Evidence from clinical and preclinical studies supporting the role of MT in IBD, in relation to the gut microbiota.

**Preclinical Studies of MT’s Microbiota-Mediated Anti-Colitic Effect**				
**Study**	**Experimental Model of Colitis**	**MT Administration**	**Outcomes of MT Treatment—Related to Microbiota**	**Other Outcomes**
[134]	5% DSS in water in ICR mice aged 8 weeks	0.2 mg/L MT in water, for one week	↑*Firmicutes/Bacteroidetes* ratio ↑*Coprococcus*_1, *Ruminococcaceae* UCG-014	↑overall antioxidant capability
[135]	2.5% (*w/v*) DSS in the drinking water for 6 days, in 8–9-week-old male WT/TLR4 KO BALB/c mice	10 mg/kg/day MT i.p. for 8 days	↑richness and diversity ↑*Firmicutes* (trend) ↓*Proteobacteria* (including *Salmonella* and *Escherichia coli*), *Bacteroidetes* (trend) ↑*Ruminococcaceae*	↓DAI ↑goblet cells, Reg3β ↓IL-1β, IL-17 (only in WT mice)
[136]	DSS	Amphiphilic conjugate of hyaluronic acid and MT	Restores the ratio of *Firmicutes/Bacteroidetes* ↑richness and diversity ↑*Lactobacillus* ↓*Bacteroides*, *Blautia*, *Streptococcus*	Improvement of the colitis symptoms Alleviation of the damaged intestinal barrier Inhibition of the colonic inflammation
[137]	4.5 mL/g of 3% oxazolone solution injected in the colon, in male C57BL/6 mice aged 6–8 weeks	50 mg/kg of MT daily for one week before induction of colitis	↑*Verrucomicrobiota*, *Actinobacteria* ↑*Bifidobacterium* ↓*Desulfovibrio*, *Lachnospiraceae*, *Peptococcaceae*	↑colon length ↓body weight loss ↓pathology score ↑occludin and ZO-1 ↓TNF-α, IL-1β, IL-5, IL-13, CD11b + Ly6G+ neutrophils
**Clinical Studies of MT in IBD**				
**Study**	**Design**	**Participants**	**MT Administration**	**Treatment Outcomes**
[138]	Comparative study	40 patients with UC/CD	30 days	↓inflammatory gut infiltration Improved intestinal ultrastructure
[139]	RCT	60 patients with left-sided UC (38 women and 22 men, aged 26–49 years)	Mesalazine in daily doses 2 × 1.0 g and MT 5 mg daily at bedtime (group I) or placebo (group II)	↑remission rate ↓MCDAI prevention of CRP increases and hemoglobin reduction

*Abbreviations:* ↑, Increase; ↓, Decrease; DSS, dextran sulfate sodium; WT, wild type; TLR, Toll-like receptor; KO, knockout; DAI, disease activity index; Reg, regenerating islet-derived protein; IL, interleukin; ZO, zonula occludens; TNF, tumor necrosis factor; UC, ulcerative colitis; CD, Crohn’s disease; RCT, randomized controlled trial; MCDAI, microscopic colitis disease activity index; CRP, C-reactive protein; SCCAI, simple clinical colitis activity index; SF-36, 36-item short form health survey.

**Table 2:** Impact of MT administration on gut microbiota composition in preclinical models of colitis.

Taxonomic Level	Microorganism	Effect of MT Administration	Role of Microorganism in IBD
Phylum	*Firmicutes/Bacteroidetes (ratio)*	Increase	Index of intestinal homeostasis; reduced in IBD
	*Proteobacteria*	Decrease	Role in IBD pathogenesis
Family	*Ruminococcaceae*	Increase	SCFA-producing; decreased in IBD
	*Peptococcaceae*	Decrease	Intestinal inflammation
	*Lachnospiraceae*	Decrease	Increased in stress-induced gut dysbiosis
Genus	*Coprococcus*	Increase	SCFA-producing; decreased in IBD
	*Bifidobacterium*	Increase	Probiotic used in IBD
	*Lactobacillus*	Increase	Probiotic used in IBD
	*Streptococcus*	Decrease	Associated with disease activity in IBD
	*Desulfovibrio*	Decrease	Associated with UC

**Table 3:** Evidence from animal studies to support the impact of melatonin on the gut microbiome and its local and systemic effects on sleep disturbance-associated colitis.

Experimental Model	Gut Microbiome Changes	Local Effects	Systemic Effects	Reference
*ICR mice*—experimental colitis (5% DSS) for 6 days; 3 days of SD; MT (i.p. 10 mg/kg for 3 days)		↓gross rectal bleeding ↓colon inflammation ↑iNOS, Wnt5a	↓weight loss ↑survival ↓inflammatory cytokines (IL-1β, IL-6, IL-17, TNF-α, INF-γ)	[181]
*CD1 mice*—3 days of SD; MT (i.p. 20 mg/kg or 40 mg/kg for 3 days)	Phylum: ↓*Firmicutes: Bacteroidetes* ratio Family: ↓*Strepococcaceae, Lachnospiraceae, Gammaproteobacteria, Moraxellaceae* ↑*Akkermansia, Bacteroides, Faecalibacterium* ↓*Aeromonas*	↑goblet cells (MUC2) ↑enterocyte proliferation ↑claudin-1, occludin, ZO-1 ↑GSH-Px, SOD, CAT, T-AOC ↓MDA ↓NF-κB pathway ↓autophagy (ATG5, Beclin1)	↓NE, IL-1β, IL-6, TNF-α ↑IL-5, IL-10, IFN-γ	[76]
*CD1 mice*—3 days of SD; MT (i.p. 20 mg/kg or 40 mg/kg for 3 days)	Phylum: ↓*Firmicutes: Bacteroidetes* ratio Family: ↑*Prevotellaceae, Bacteroidaceae* ↓*Moraxellaceae, Aeromonadaceae, Rikenellaceae, Ruminococcaceae*, *Gammaproteobacteria* Genus: ↓*Aeromonas*	↓ROS ↓IL-17 ↑IL-22	↓Cort	[77]
*C57BL/6 mice*—10 days of partial SD (6 h during light cycle); MT (i.p. 10 mg/kg for 10 days)	Family: ↓*Erysipelotrichaceae* Species: ↑*Akkermansia muciniphila, Lactobacillus murinus* ↓*Bacteroides massiliensis, Enterobacter cloacae, Enterobacter asburia*			[21]
*CD1 mice*—3 days of SD; MT (i.p. 20 mg/kg for 3 days) w/o FMT	Phylum: ↓*Firmicutes:Bacteroidetes* ratio ↓*Proteobacteria* Family: ↑*Prevotellaceae*	↑goblet cells (MUC2) ↑claudin-1, ZO-1 ↑CARD9 ↓IL-17 ↓ROS ↓HSP90 ↑HSP70, P23 ↓STAT3/AP-1/NF-kB	↓ DAI ↓Cort, GR	[182]
*ICR mice*—3 days of SD; MT (i.p. 20 mg/kg for 3 days) w/o AB cocktail, 40 mM butyrate, FMT	Phylum: ↓*Firmicutes:Bacteroidetes* ratio ↓*Proteobacteria* ↑*Verrucomicrobia* Genus: ↑*Faecalibacterium-*↑butyrate (↑MCT1)	↓IL-1β , IL-6, TNF-α, IL-17 ↑IL-10, IFN-γ ↓NF-kB/NLRP3 loop ↓HDAC3-↑p-GSK-3β/β-catenin/HIF-1α ↑ CARD9		[20]
*C57BL/6J mice*—JL induction; MT in drinking water (0.4 mg/mL); AB for 10 days	Family: ↓*Enterobacteriales* Species: ↑*Akkermansia muciniphila* ↓*Escherichia coli*	↓LPCAT3, FATP4, NPC1L1, CD36 (associated with ileal lipid intake) ↓ fat accumulation in eWAT		[84]
*ICR mice*—3 days of SD; MT (i.p. 20 mg/kg for 3 days), FMT, w/o 10^8^ CFU (*Aeromonas veronii*), LPS (i.p., 2 mg/kg) w/o TAK-242 (TLR4 inhibitor)	Phylum: ↓*Firmicutes: Bacteroidetes* ratio Species: ↓*Aeromonas veronii*	↓colon shortening ↓fecal occult blood ↓intestinal permeability ↑goblet cells (MUC2, Villin, Tff3) ↓ROS ↓IL-1β, TNF-α, IL-17 ↑IL-10, IFN-γ ↓NF-kB/NLRP3 ↓TLR4/MyD88/GSK-3β/β-catenin/NF-κB loop	↓weight loss ↓DAI	[183]
*CD1 mice*—28 days of SR (4 h/day); MT (10^−5^ mol/L in drinking water)	Phylum: ↓*Firmicutes: Bacteroidetes* ratio Genus: ↑*Lactobacillus* ↓*Helicobacter, Clostridium*	↓ IL-6, TNF-α ↑ IL-10, IFN-γ ↑GSH-Px, SOD, CAT, T-AOC ↓MDA	↓NE, Cort, Glucose	[175]

*Abbreviations:* ↑, Increase; ↓, Decrease; MT-melatonin, SD-sleep deprivation, ICR—Imprinting control region, iNOS—inducible nitric oxide synthase, Wnt5a—wingless-type MMTV integration site family, SR—sleep rectriction, CAT-catalase, SOD—superoxid dismutase, NF-κB—Nuclear factor kappa-light-chain-enhancer of activated B cells, i.p.—intraperitoneal, DSS—dextran sulfate sodium, TNF-α—tumor necrosis factor- α, IL—interleukin, IFN-γ—interferon γ, GSH-Px—glutathione peroxidase, SOD—superoxide dismutase, CAT—catalase, T-AOC—total antioxidant capability, MDA—malondialdehyde, ZO-1—zonula occludens-1, Cort—corticosterone, ROS—reactive oxygen species, FMT—fecal microbiota transplantation, AB—antibiotic, DAI—disease activity index, CARD9—Caspase Recruitment Domain Family Member 9, HSP—heat shock protein, GR—glucocorticoid receptor, STAT—Signal Transducer and Activator of Transcription, AP-1—activator protein-1, JL—jetlag, MCT1—monocarboxylate-transporter 1, NLRP-3—Nucleotide-binding oligomerization domain, Leucine rich Repeat and Pyrin domain containing Proteins, p-GSK-3β—Glycogen synthase kinase-3, HIF-1α—Hypoxia-Inducible Factor-1α, HDAC3—Histone Deacetylase 3, ANGPTL4—Angiopoietin-like 4, eWAT—white adipose tissue, LPS—lipopolysaccharide, TLR4—Toll-like receptor, LPCAT1—Lysophosphatidylcholine acyltransferase 1, FATP4—fatty acid transporter protein4, NPC1L1—NPC1-like intracellular cholesterol transporter 1, CD36—cluster of differentiation 36, Tff3—Trefoil Factor 3, MyD88—Myeloid differentiation primary response 88, —norepinephrine.

## Data Availability

All the date is contained within the article.

## References

[1] Cipolla-Neto J., Amaral F.G. (2018). do Melatonin as a Hormone: New Physiological and Clinical Insights. Endocr. Rev..

[2] Lerner A.B., Case J.D., Takahashi Y., Lee T.H., Mori W. (1958). Isolation of Melatonin, the Pineal Gland Factor That Lightens Melanocytes. J. Am. Chem. Soc..

[3] Gitto E., Aversa S., Reiter R.J., Barberi I., Pellegrino S. (2011). Update on the Use of Melatonin in Pediatrics. J. Pineal Res..

[4] Andersen L.P.H., Werner M.U., Rosenberg J., Gögenur I. (2014). A Systematic Review of Peri-Operative Melatonin. Anaesthesia.

[5] Arendt J., Skene D.J. (2005). Melatonin as a Chronobiotic. Sleep Med. Rev..

[6] Andersen L.P.H., Gögenur I., Rosenberg J., Reiter R.J. (2016). The Safety of Melatonin in Humans. Clin. Drug Investig..

[7] Tan D.-X., Manchester L.C., Terron M.P., Flores L.J., Reiter R.J. (2007). One Molecule, Many Derivatives: A Never-Ending Interaction of Melatonin with Reactive Oxygen and Nitrogen Species?. J. Pineal Res..

[8] Chitimus D.M., Popescu M.R., Voiculescu S.E., Panaitescu A.M., Pavel B., Zagrean L., Zagrean A.-M. (2020). Melatonin’s Impact on Antioxidative and Anti-Inflammatory Reprogramming in Homeostasis and Disease. Biomolecules.

[9] Acuña-Castroviejo D., Escames G., Venegas C., Díaz-Casado M.E., Lima-Cabello E., López L.C., Rosales-Corral S., Tan D.-X., Reiter R.J. (2014). Extrapineal Melatonin: Sources, Regulation, and Potential Functions. Cell. Mol. Life Sci..

[10] Kvetnoy I.M., Ingel I.E., Kvetnaia T.V., Malinovskaya N.K., Rapoport S.I., Raikhlin N.T., Trofimov A.V., Yuzhakov V.V. (2002). Gastrointestinal Melatonin: Cellular Identification and Biological Role. Neuro Endocrinol. Lett..

[11] Novais A.A., Chuffa L.G.d.A., Zuccari D.A.P.d.C., Reiter R.J. (2021). Exosomes and Melatonin: Where Their Destinies Intersect. Front. Immunol..

[12] Bubenik G.A. (2001). Localization, Physiological Significance and Possible Clinical Implication of Gastrointestinal Melatonin. Biol. Signals Recept.

[13] Bubenik G.A., Brown G.M., Grota L.J. (1977). Immunohistological Localization of Melatonin in the Rat Digestive System. Experientia.

[14] Bubenik G.A., Niles L.P., Pang S.F., Pentney P.J. (1993). Diurnal Variation and Binding Characteristics of Melatonin in the Mouse Brain and Gastrointestinal Tissues. Comp. Biochem. Physiol. C Comp. Pharm. Toxicol..

[15] Bubenik G.A., Hacker R.R., Brown G.M., Bartos L. (1999). Melatonin Concentrations in the Luminal Fluid, Mucosa, and Muscularis of the Bovine and Porcine Gastrointestinal Tract. J. Pineal Res..

[16] Chen C.-Q., Fichna J., Bashashati M., Li Y.-Y., Storr M. (2011). Distribution, Function and Physiological Role of Melatonin in the Lower Gut. World J. Gastroenterol..

[17] Bull M.J., Plummer N.T. (2014). Part 1: The Human Gut Microbiome in Health and Disease. Integr. Med..

[18] Cryan J.F., Dinan T.G. (2012). Mind-Altering Microorganisms: The Impact of the Gut Microbiota on Brain and Behaviour. Nat. Rev. Neurosci..

[19] Yasmin F., Sutradhar S., Das P., Mukherjee S. (2021). Gut Melatonin: A Potent Candidate in the Diversified Journey of Melatonin Research. Gen. Comp. Endocrinol..

[20] Gao T., Wang Z., Dong Y., Cao J., Chen Y. (2021). Melatonin-Mediated Colonic Microbiota Metabolite Butyrate Prevents Acute Sleep Deprivation-Induced Colitis in Mice. Int. J. Mol. Sci..

[21] Park Y.S., Kim S.H., Park J.W., Kho Y., Seok P.R., Shin J.-H., Choi Y.J., Jun J.-H., Jung H.C., Kim E.K. (2020). Melatonin in the Colon Modulates Intestinal Microbiota in Response to Stress and Sleep Deprivation. Intest. Res..

[22] Wilkins L.J., Monga M., Miller A.W. (2019). Defining Dysbiosis for a Cluster of Chronic Diseases. Sci. Rep..

[23] Stefulj J., Hörtner M., Ghosh M., Schauenstein K., Rinner I., Wölfler A., Semmler J., Liebmann P.M. (2001). Gene Expression of the Key Enzymes of Melatonin Synthesis in Extrapineal Tissues of the Rat. J. Pineal Res..

[24] Hong G.X., Pang S.F. (1995). N-Acetyltransferase Activity in the Quail (Coturnix Coturnix Jap) Duodenum. Comp. Biochem. Physiol. B Biochem. Mol. Biol..

[25] Bubenik G.A., Brown G.M. (1997). Pinealectomy Reduces Melatonin Levels in the Serum but Not in the Gastrointestinal Tract of Rats. Biol. Signals.

[26] Bubenik G.A., Pang S.F. (1997). Melatonin Levels in the Gastrointestinal Tissues of Fish, Amphibians, and a Reptile. Gen. Comp. Endocrinol..

[27] Messner M., Huether G., Lorf T., Ramadori G., Schwörer H. (2001). Presence of Melatonin in the Human Hepatobiliary-Gastrointestinal Tract. Life Sci..

[28] Vician M., Zeman M., Herichová I., Juráni M., Blazícek P., Matis P. (1999). Melatonin Content in Plasma and Large Intestine of Patients with Colorectal Carcinoma before and after Surgery. J. Pineal Res..

[29] Pontoire C., Bernard M., Silvain C., Collin J.P., Voisin P. (1993). Characterization of Melatonin Binding Sites in Chicken and Human Intestines. Eur. J. Pharm..

[30] Poon A.M., Chow P.H., Mak A.S., Pang S.F. (1997). Autoradiographic Localization of 2[125I]Iodomelatonin Binding Sites in the Gastrointestinal Tract of Mammals Including Humans and Birds. J. Pineal Res..

[31] Pal P.K., Sarkar S., Chattopadhyay A., Tan D.X., Bandyopadhyay D. (2019). Enterochromaffin Cells as the Source of Melatonin: Key Findings and Functional Relevance in Mammals. Melatonin Res..

[32] Rezzani R., Franco C., Franceschetti L., Gianò M., Favero G. (2022). A Focus on Enterochromaffin Cells among the Enteroendocrine Cells: Localization, Morphology, and Role. Int. J. Mol. Sci..

[33] Söderquist F., Hellström P.M., Cunningham J.L. (2015). Human Gastroenteropancreatic Expression of Melatonin and Its Receptors MT1 and MT2. PLoS ONE.

[34] Richard D.M., Dawes M.A., Mathias C.W., Acheson A., Hill-Kapturczak N., Dougherty D.M. (2009). L-Tryptophan: Basic Metabolic Functions, Behavioral Research and Therapeutic Indications. Int. J. Tryptophan. Res..

[35] Sikander A., Rana S.V., Prasad K.K. (2009). Role of Serotonin in Gastrointestinal Motility and Irritable Bowel Syndrome. Clin. Chim. Acta.

[36] Agus A., Planchais J., Sokol H. (2018). Gut Microbiota Regulation of Tryptophan Metabolism in Health and Disease. Cell Host Microbe.

[37] Gao K., Mu C.-L., Farzi A., Zhu W.-Y. (2020). Tryptophan Metabolism: A Link Between the Gut Microbiota and Brain. Adv. Nutr..

[38] Li Y., Liu N., Ge Y., Yang Y., Ren F., Wu Z. (2022). Tryptophan and the Innate Intestinal Immunity: Crosstalk between Metabolites, Host Innate Immune Cells, and Microbiota. Eur. J. Immunol..

[39] Taleb S. (2019). Tryptophan Dietary Impacts Gut Barrier and Metabolic Diseases. Front. Immunol..

[40] Meng X., Li Y., Li S., Zhou Y., Gan R.-Y., Xu D.-P., Li H.-B. (2017). Dietary Sources and Bioactivities of Melatonin. Nutrients.

[41] von Gall C., Stehle J.H., Weaver D.R. (2002). Mammalian Melatonin Receptors: Molecular Biology and Signal Transduction. Cell Tissue Res..

[42] Bondi C.D., McKeon R.M., Bennett J.M., Ignatius P.F., Brydon L., Jockers R., Melan M.A., Witt-Enderby P.A. (2008). MT1 Melatonin Receptor Internalization Underlies Melatonin-Induced Morphologic Changes in Chinese Hamster Ovary Cells and These Processes Are Dependent on Gi Proteins, MEK 1/2 and Microtubule Modulation. J. Pineal Res..

[43] Chan A.S.L., Lai F.P.L., Lo R.K.H., Voyno-Yasenetskaya T.A., Stanbridge E.J., Wong Y.H. (2002). Melatonin Mt1 and MT2 Receptors Stimulate C-Jun N-Terminal Kinase via Pertussis Toxin-Sensitive and -Insensitive G Proteins. Cell. Signal..

[44] Ho M.K., Yung L.Y., Chan J.S., Chan J.H., Wong C.S., Wong Y.H. (2001). Galpha(14) Links a Variety of G(i)- and G(s)-Coupled Receptors to the Stimulation of Phospholipase C. Br. J. Pharm..

[45] Jockers R., Maurice P., Boutin J.A., Delagrange P. (2008). Melatonin Receptors, Heterodimerization, Signal Transduction and Binding Sites: What’s New?. Br. J. Pharm..

[46] Wang R.-X., Liu H., Xu L., Zhang H., Zhou R.-X. (2015). Involvement of Nuclear Receptor RZR/RORγ in Melatonin-Induced HIF-1α Inactivation in SGC-7901 Human Gastric Cancer Cells. Oncol. Rep..

[47] Winczyk K., Pawlikowski M., Karasek M. (2001). Melatonin and RZR/ROR Receptor Ligand CGP 52608 Induce Apoptosis in the Murine Colonic Cancer. J. Pineal Res..

[48] Aydin M., Canpolat S., Kuloğlu T., Yasar A., Colakoglu N., Kelestimur H. (2008). Effects of Pinealectomy and Exogenous Melatonin on Ghrelin and Peptide YY in Gastrointestinal System and Neuropeptide Y in Hypothalamic Arcuate Nucleus: Immunohistochemical Studies in Male Rats. Regul. Pept..

[49] Jaworek J., Brzozowski T., Konturek S.J. (2005). Melatonin as an Organoprotector in the Stomach and the Pancreas. J. Pineal Res..

[50] Sjöblom M., Flemström G. (2003). Melatonin in the Duodenal Lumen Is a Potent Stimulant of Mucosal Bicarbonate Secretion. J. Pineal Res..

[51] Thursby E., Juge N. (2017). Introduction to the Human Gut Microbiota. Biochem. J..

[52] Bäckhed F., Roswall J., Peng Y., Feng Q., Jia H., Kovatcheva-Datchary P., Li Y., Xia Y., Xie H., Zhong H. (2015). Dynamics and Stabilization of the Human Gut Microbiome during the First Year of Life. Cell Host Microbe.

[53] Hasan N., Yang H. (2019). Factors Affecting the Composition of the Gut Microbiota, and Its Modulation. PeerJ.

[54] Turnbaugh P.J., Ridaura V.K., Faith J.J., Rey F.E., Knight R., Gordon J.I. (2009). The Effect of Diet on the Human Gut Microbiome: A Metagenomic Analysis in Humanized Gnotobiotic Mice. Sci. Transl. Med..

[55] Ramirez J., Guarner F., Bustos Fernandez L., Maruy A., Sdepanian V.L., Cohen H. (2020). Antibiotics as Major Disruptors of Gut Microbiota. Front. Cell. Infect. Microbiol..

[56] O’Mahony S.M., Marchesi J.R., Scully P., Codling C., Ceolho A.-M., Quigley E.M.M., Cryan J.F., Dinan T.G. (2009). Early Life Stress Alters Behavior, Immunity, and Microbiota in Rats: Implications for Irritable Bowel Syndrome and Psychiatric Illnesses. Biol. Psychiatry.

[57] Rinninella E., Raoul P., Cintoni M., Franceschi F., Miggiano G.A.D., Gasbarrini A., Mele M.C. (2019). What Is the Healthy Gut Microbiota Composition? A Changing Ecosystem across Age, Environment, Diet, and Diseases. Microorganisms.

[58] Qin J., Li R., Raes J., Arumugam M., Burgdorf K.S., Manichanh C., Nielsen T., Pons N., Levenez F., Yamada T. (2010). A Human Gut Microbial Gene Catalog Established by Metagenomic Sequencing. Nature.

[59] Stojanov S., Berlec A., Štrukelj B. (2020). The Influence of Probiotics on the Firmicutes/Bacteroidetes Ratio in the Treatment of Obesity and Inflammatory Bowel Disease. Microorganisms.

[60] Shin N.-R., Whon T.W., Bae J.-W. (2015). Proteobacteria: Microbial Signature of Dysbiosis in Gut Microbiota. Trends Biotechnol..

[61] Young V.B., Schmidt T.M. (2008). Overview of the Gastrointestinal Microbiota. Adv. Exp. Med. Biol..

[62] Tan J., McKenzie C., Potamitis M., Thorburn A.N., Mackay C.R., Macia L. (2014). The Role of Short-Chain Fatty Acids in Health and Disease. Adv. Immunol..

[63] Pascale A., Marchesi N., Marelli C., Coppola A., Luzi L., Govoni S., Giustina A., Gazzaruso C. (2018). Microbiota and Metabolic Diseases. Endocrine.

[64] Fung T.C., Olson C.A., Hsiao E.Y. (2017). Interactions between the Microbiota, Immune and Nervous Systems in Health and Disease. Nat. Neurosci..

[65] Arpaia N., Campbell C., Fan X., Dikiy S., van der Veeken J., deRoos P., Liu H., Cross J.R., Pfeffer K., Coffer P.J. (2013). Metabolites Produced by Commensal Bacteria Promote Peripheral Regulatory T-Cell Generation. Nature.

[66] Smith P.M., Howitt M.R., Panikov N., Michaud M., Gallini C.A., Bohlooly-Y M., Glickman J.N., Garrett W.S. (2013). The Microbial Metabolites, Short-Chain Fatty Acids, Regulate Colonic Treg Cell Homeostasis. Science.

[67] Ahlawat S., Kumar P., Mohan H., Goyal S., Sharma K.K. (2021). Inflammatory Bowel Disease: Tri-Directional Relationship between Microbiota, Immune System and Intestinal Epithelium. Crit. Rev. Microbiol..

[68] Santana P.T., Rosas S.L.B., Ribeiro B.E., Marinho Y., de Souza H.S.P. (2022). Dysbiosis in Inflammatory Bowel Disease: Pathogenic Role and Potential Therapeutic Targets. Int. J. Mol. Sci..

[69] Frank D.N., St Amand A.L., Feldman R.A., Boedeker E.C., Harpaz N., Pace N.R. (2007). Molecular-Phylogenetic Characterization of Microbial Community Imbalances in Human Inflammatory Bowel Diseases. Proc. Natl. Acad. Sci. USA.

[70] Sultan S., El-Mowafy M., Elgaml A., Ahmed T.A.E., Hassan H., Mottawea W. (2021). Metabolic Influences of Gut Microbiota Dysbiosis on Inflammatory Bowel Disease. Front. Physiol..

[71] Oka A., Sartor R.B. (2020). Microbial-Based and Microbial-Targeted Therapies for Inflammatory Bowel Diseases. Dig. Dis. Sci..

[72] Cenit M.C., Sanz Y., Codoñer-Franch P. (2017). Influence of Gut Microbiota on Neuropsychiatric Disorders. World J. Gastroenterol..

[73] Chojnacki C., Popławski T., Blasiak J., Chojnacki J., Reiter R.J., Klupinska G. (2013). Expression of Melatonin Synthesizing Enzymes in Helicobacter Pylori Infected Gastric Mucosa. BioMed Res. Int..

[74] Wong R.K., Yang C., Song G.-H., Wong J., Ho K.-Y. (2015). Melatonin Regulation as a Possible Mechanism for Probiotic (VSL#3) in Irritable Bowel Syndrome: A Randomized Double-Blinded Placebo Study. Dig. Dis. Sci..

[75] Lutfi E., Basili D., Falcinelli S., Morillas L., Carnevali O., Capilla E., Navarro I. (2021). The Probiotic Lactobacillus Rhamnosus Mimics the Dark-Driven Regulation of Appetite Markers and Melatonin Receptors’ Expression in Zebrafish (Danio Rerio) Larvae: Understanding the Role of the Gut Microbiome. Comp. Biochem. Physiol. Part B Biochem. Mol. Biol..

[76] Gao T., Wang Z., Dong Y., Cao J., Lin R., Wang X., Yu Z., Chen Y. (2019). Role of Melatonin in Sleep Deprivation-Induced Intestinal Barrier Dysfunction in Mice. J. Pineal Res..

[77] Gao T., Wang Z., Cao J., Dong Y., Chen Y. (2020). Melatonin Attenuates Microbiota Dysbiosis of Jejunum in Short-Term Sleep Deprived Mice. J. Microbiol..

[78] Zhang L., Guadarrama L., Corona-Morales A.A., Vega-Gonzalez A., Rocha L., Escobar A. (2006). Rats Subjected to Extended L-Tryptophan Restriction during Early Postnatal Stage Exhibit Anxious-Depressive Features and Structural Changes. J. Neuropathol. Exp. Neurol..

[79] Wikoff W.R., Anfora A.T., Liu J., Schultz P.G., Lesley S.A., Peters E.C., Siuzdak G. (2009). Metabolomics Analysis Reveals Large Effects of Gut Microflora on Mammalian Blood Metabolites. Proc. Natl. Acad. Sci. USA.

[80] Choi S.-C., Brown J., Gong M., Ge Y., Zadeh M., Li W., Croker B.P., Michailidis G., Garrett T.J., Mohamadzadeh M. (2020). Gut Microbiota Dysbiosis and Altered Tryptophan Catabolism Contribute to Autoimmunity in Lupus-Susceptible Mice. Sci. Transl. Med..

[81] Yano J.M., Yu K., Donaldson G.P., Shastri G.G., Ann P., Ma L., Nagler C.R., Ismagilov R.F., Mazmanian S.K., Hsiao E.Y. (2015). Indigenous Bacteria from the Gut Microbiota Regulate Host Serotonin Biosynthesis. Cell.

[82] Yildirim A., Arabacı Tamer S., Sahin D., Bagriacik F., Kahraman M.M., Onur N.D., Cayirli Y.B., Cilingir Kaya Ö.T., Aksu B., Akdeniz E. (2019). The Effects of Antibiotics and Melatonin on Hepato-Intestinal Inflammation and Gut Microbial Dysbiosis Induced by a Short-Term High-Fat Diet Consumption in Rats. Br. J. Nutr..

[83] Zhang Z., Peng Q., Huo D., Jiang S., Ma C., Chang H., Chen K., Li C., Pan Y., Zhang J. (2021). Melatonin Regulates the Neurotransmitter Secretion Disorder Induced by Caffeine Through the Microbiota-Gut-Brain Axis in Zebrafish (Danio Rerio). Front. Cell. Dev. Biol..

[84] Rong B., Wu Q., Reiter R.J., Sun C. (2021). The Mechanism of Oral Melatonin Ameliorates Intestinal and Adipose Lipid Dysmetabolism Through Reducing Escherichia Coli-Derived Lipopolysaccharide. Cell. Mol. Gastroenterol. Hepatol..

[85] Tordjman S., Chokron S., Delorme R., Charrier A., Bellissant E., Jaafari N., Fougerou C. (2017). Melatonin: Pharmacology, Functions and Therapeutic Benefits. Curr. Neuropharmacol..

[86] Hastings M.H., Maywood E.S., Brancaccio M. (2019). The Mammalian Circadian Timing System and the Suprachiasmatic Nucleus as Its Pacemaker. Biology.

[87] Lee H.S., Billings H.J., Lehman M.N. (2003). The Suprachiasmatic Nucleus: A Clock of Multiple Components. J. Biol. Rhythm..

[88] Warren E.J., Allen C.N., Brown R.L., Robinson D.W. (2006). The Light-Activated Signaling Pathway in SCN-Projecting Rat Retinal Ganglion Cells. Eur. J. Neurosci..

[89] Xie Y., Tang Q., Chen G., Xie M., Yu S., Zhao J., Chen L. (2019). New Insights Into the Circadian Rhythm and Its Related Diseases. Front. Physiol..

[90] Liu C., Weaver D.R., Jin X., Shearman L.P., Pieschl R.L., Gribkoff V.K., Reppert S.M. (1997). Molecular Dissection of Two Distinct Actions of Melatonin on the Suprachiasmatic Circadian Clock. Neuron.

[91] Hunt A.E., Al-Ghoul W.M., Gillette M.U., Dubocovich M.L. (2001). Activation of MT(2) Melatonin Receptors in Rat Suprachiasmatic Nucleus Phase Advances the Circadian Clock. Am. J. Physiol. Cell. Physiol..

[92] Agez L., Laurent V., Pévet P., Masson-Pévet M., Gauer F. (2007). Melatonin Affects Nuclear Orphan Receptors MRNA in the Rat Suprachiasmatic Nuclei. Neuroscience.

[93] Pévet P., Agez L., Bothorel B., Saboureau M., Gauer F., Laurent V., Masson-Pévet M. (2006). Melatonin in the Multi-Oscillatory Mammalian Circadian World. Chronobiol. Int..

[94] Valenzuela F.J., Torres-Farfan C., Richter H.G., Mendez N., Campino C., Torrealba F., Valenzuela G.J., Serón-Ferré M. (2008). Clock Gene Expression in Adult Primate Suprachiasmatic Nuclei and Adrenal: Is the Adrenal a Peripheral Clock Responsive to Melatonin?. Endocrinology.

[95] Zeman M., Herichova I. (2013). Melatonin and Clock Genes Expression in the Cardiovascular System. Front. Biosci..

[96] Sandu C., Liu T., Malan A., Challet E., Pévet P., Felder-Schmittbuhl M.-P. (2015). Circadian Clocks in Rat Skin and Dermal Fibroblasts: Differential Effects of Aging, Temperature and Melatonin. Cell. Mol. Life Sci..

[97] Mukherji A., Kobiita A., Ye T., Chambon P. (2013). Homeostasis in Intestinal Epithelium Is Orchestrated by the Circadian Clock and Microbiota Cues Transduced by TLRs. Cell.

[98] Zarrinpar A., Chaix A., Yooseph S., Panda S. (2014). Diet and Feeding Pattern Affect the Diurnal Dynamics of the Gut Microbiome. Cell Metab..

[99] Thaiss C.A., Zeevi D., Levy M., Zilberman-Schapira G., Suez J., Tengeler A.C., Abramson L., Katz M.N., Korem T., Zmora N. (2014). Transkingdom Control of Microbiota Diurnal Oscillations Promotes Metabolic Homeostasis. Cell.

[100] Fowler S., Hoedt E.C., Talley N.J., Keely S., Burns G.L. (2022). Circadian Rhythms and Melatonin Metabolism in Patients With Disorders of Gut-Brain Interactions. Front. Neurosci..

[101] Thaiss C.A., Levy M., Korem T., Dohnalová L., Shapiro H., Jaitin D.A., David E., Winter D.R., Gury-BenAri M., Tatirovsky E. (2016). Microbiota Diurnal Rhythmicity Programs Host Transcriptome Oscillations. Cell.

[102] Paulose J.K., Wright J.M., Patel A.G., Cassone V.M. (2016). Human Gut Bacteria Are Sensitive to Melatonin and Express Endogenous Circadian Rhythmicity. PLoS ONE.

[103] Heddes M., Altaha B., Niu Y., Reitmeier S., Kleigrewe K., Haller D., Kiessling S. (2022). The Intestinal Clock Drives the Microbiome to Maintain Gastrointestinal Homeostasis. Nat. Commun..

[104] Parkar S.G., Kalsbeek A., Cheeseman J.F. (2019). Potential Role for the Gut Microbiota in Modulating Host Circadian Rhythms and Metabolic Health. Microorganisms.

[105] Leone V., Gibbons S.M., Martinez K., Hutchison A.L., Huang E.Y., Cham C.M., Pierre J.F., Heneghan A.F., Nadimpalli A., Hubert N. (2015). Effects of Diurnal Variation of Gut Microbes and High-Fat Feeding on Host Circadian Clock Function and Metabolism. Cell Host Microbe.

[106] Kolbe I., Oster H. (2019). Chronodisruption, Metabolic Homeostasis, and the Regulation of Inflammation in Adipose Tissues. Yale J. Biol. Med..

[107] Murakami M., Tognini P. (2020). The Circadian Clock as an Essential Molecular Link Between Host Physiology and Microorganisms. Front. Cell. Infect. Microbiol..

[108] Yin J., Li Y., Han H., Ma J., Liu G., Wu X., Huang X., Fang R., Baba K., Bin P. (2020). Administration of Exogenous Melatonin Improves the Diurnal Rhythms of the Gut Microbiota in Mice Fed a High-Fat Diet. mSystems.

[109] Hardeland R., Reiter R.J., Poeggeler B., Tan D.X. (1993). The Significance of the Metabolism of the Neurohormone Melatonin: Antioxidative Protection and Formation of Bioactive Substances. Neurosci. Biobehav. Rev..

[110] Hardeland R., Balzer I., Poeggeler B., Fuhrberg B., Uría H., Behrmann G., Wolf R., Meyer T.J., Reiter R.J. (1995). On the Primary Functions of Melatonin in Evolution: Mediation of Photoperiodic Signals in a Unicell, Photooxidation, and Scavenging of Free Radicals. J. Pineal Res..

[111] Allegra M., Reiter R.J., Tan D.-X., Gentile C., Tesoriere L., Livrea M.A. (2003). The Chemistry of Melatonin’s Interaction with Reactive Species. J. Pineal Res..

[112] Reiter R.J., Tan D.X., Osuna C., Gitto E. (2000). Actions of Melatonin in the Reduction of Oxidative Stress. A Review. J. Biomed. Sci..

[113] Rodriguez C., Mayo J.C., Sainz R.M., Antolín I., Herrera F., Martín V., Reiter R.J. (2004). Regulation of Antioxidant Enzymes: A Significant Role for Melatonin. J. Pineal Res..

[114] Urata Y., Honma S., Goto S., Todoroki S., Iida T., Cho S., Honma K., Kondo T. (1999). Melatonin Induces Gamma-Glutamylcysteine Synthetase Mediated by Activator Protein-1 in Human Vascular Endothelial Cells. Free Radic. Biol. Med..

[115] Gitto E., Tan D.X., Reiter R.J., Karbownik M., Manchester L.C., Cuzzocrea S., Fulia F., Barberi I. (2001). Individual and Synergistic Antioxidative Actions of Melatonin: Studies with Vitamin E, Vitamin C, Glutathione and Desferrioxamine (Desferoxamine) in Rat Liver Homogenates. J. Pharm. Pharm..

[116] Singh V., Ahlawat S., Mohan H., Gill S.S., Sharma K.K. (2022). Balancing Reactive Oxygen Species Generation by Rebooting Gut Microbiota. J. Appl. Microbiol..

[117] Sies H. (2015). Oxidative Stress: A Concept in Redox Biology and Medicine. Redox Biol..

[118] Le Gal K., Schmidt E.E., Sayin V.I. (2021). Cellular Redox Homeostasis. Antioxidants.

[119] Jones R.M., Mercante J.W., Neish A.S. (2012). Reactive Oxygen Production Induced by the Gut Microbiota: Pharmacotherapeutic Implications. Curr. Med. Chem..

[120] Ni Q., Zhang P., Li Q., Han Z. (2022). Oxidative Stress and Gut Microbiome in Inflammatory Skin Diseases. Front. Cell. Dev. Biol..

[121] Dumitrescu L., Popescu-Olaru I., Cozma L., Tulbă D., Hinescu M.E., Ceafalan L.C., Gherghiceanu M., Popescu B.O. (2018). Oxidative Stress and the Microbiota-Gut-Brain Axis. Oxid. Med. Cell. Longev..

[122] Yardeni T., Tanes C.E., Bittinger K., Mattei L.M., Schaefer P.M., Singh L.N., Wu G.D., Murdock D.G., Wallace D.C. (2019). Host Mitochondria Influence Gut Microbiome Diversity: A Role for ROS. Sci. Signal..

[123] Carrillo-Vico A., Lardone P.J., Alvarez-Sánchez N., Rodríguez-Rodríguez A., Guerrero J.M. (2013). Melatonin: Buffering the Immune System. Int. J. Mol. Sci..

[124] Guerrero J.M., Reiter R.J. (2002). Melatonin-Immune System Relationships. Curr. Top Med. Chem..

[125] Currier N.L., Sun L.Z., Miller S.C. (2000). Exogenous Melatonin: Quantitative Enhancement in Vivo of Cells Mediating Non-Specific Immunity. J. Neuroimmunol..

[126] Peña C., Rincon J., Pedreanez A., Viera N., Mosquera J. (2007). Chemotactic Effect of Melatonin on Leukocytes. J. Pineal Res..

[127] Inserra P., Zhang Z., Ardestani S.K., Araghi-Niknam M., Liang B., Jiang S., Shaw D., Molitor M., Elliott K., Watson R.R. (1998). Modulation of Cytokine Production by Dehydroepiandrosterone (DHEA) plus Melatonin (MLT) Supplementation of Old Mice. Proc. Soc. Exp. Biol. Med..

[128] Jaworek J., Szklarczyk J., Jaworek A.K., Nawrot-Porąbka K., Leja-Szpak A., Bonior J., Kot M. (2012). Protective Effect of Melatonin on Acute Pancreatitis. Int. J. Inflam..

[129] Veneroso C., Tuñón M.J., González-Gallego J., Collado P.S. (2009). Melatonin Reduces Cardiac Inflammatory Injury Induced by Acute Exercise. J. Pineal Res..

[130] Lin X.-J., Mei G.-P., Liu J., Li Y.-L., Zuo D., Liu S.-J., Zhao T.-B., Lin M.-T. (2011). Therapeutic Effects of Melatonin on Heatstroke-Induced Multiple Organ Dysfunction Syndrome in Rats. J. Pineal Res..

[131] Tak P.P., Firestein G.S. (2001). NF-KappaB: A Key Role in Inflammatory Diseases. J. Clin. Investig..

[132] Xu L., Zhang W., Kwak M., Zhang L., Lee P.C.W., Jin J.-O. (2019). Protective Effect of Melatonin Against Polymicrobial Sepsis Is Mediated by the Anti-Bacterial Effect of Neutrophils. Front. Immunol..

[133] He F., Wu X., Zhang Q., Li Y., Ye Y., Li P., Chen S., Peng Y., Hardeland R., Xia Y. (2021). Bacteriostatic Potential of Melatonin: Therapeutic Standing and Mechanistic Insights. Front. Immunol..

[134] Zhu D., Ma Y., Ding S., Jiang H., Fang J. (2018). Effects of Melatonin on Intestinal Microbiota and Oxidative Stress in Colitis Mice. BioMed Res. Int..

[135] Kim S.W., Kim S., Son M., Cheon J.H., Park Y.S. (2020). Melatonin Controls Microbiota in Colitis by Goblet Cell Differentiation and Antimicrobial Peptide Production through Toll-like Receptor 4 Signalling. Sci. Rep..

[136] Jing W., Zhu M., Wang F., Zhao X., Dong S., Xu Y., Wang S., Yang J., Wang K., Liu W. (2022). Hyaluronic Acid-Melatonin Nanoparticles Improve the Dysregulated Intestinal Barrier, Microbiome and Immune Response in Mice with Dextran Sodium Sulfate-Induced Colitis. J. Biomed Nanotechnol..

[137] Kowalska-Duplaga K., Gosiewski T., Kapusta P., Sroka-Oleksiak A., Wędrychowicz A., Pieczarkowski S., Ludwig-Słomczyńska A.H., Wołkow P.P., Fyderek K. (2019). Differences in the Intestinal Microbiome of Healthy Children and Patients with Newly Diagnosed Crohn’s Disease. Sci. Rep..

[138] Alam M.T., Amos G.C.A., Murphy A.R.J., Murch S., Wellington E.M.H., Arasaradnam R.P. (2020). Microbial Imbalance in Inflammatory Bowel Disease Patients at Different Taxonomic Levels. Gut Pathog..

[139] Wang Y., Gao X., Ghozlane A., Hu H., Li X., Xiao Y., Li D., Yu G., Zhang T. (2018). Characteristics of Faecal Microbiota in Paediatric Crohn’s Disease and Their Dynamic Changes During Infliximab Therapy. J. Crohn’s Colitis.

[140] Zhao Z.-X., Yuan X., Cui Y.-Y., Liu J., Shen J., Jin B.-Y., Feng B.-C., Zhai Y.-J., Zheng M.-Q., Kou G.-J. (2021). Melatonin Mitigates Oxazolone-Induced Colitis in Microbiota-Dependent Manner. Front. Immunol..

[141] Jakubczyk D., Leszczyńska K., Górska S. (2020). The Effectiveness of Probiotics in the Treatment of Inflammatory Bowel Disease (IBD)-A Critical Review. Nutrients.

[142] Wang H., Zhou C., Huang J., Kuai X., Shao X. (2020). The Potential Therapeutic Role of Lactobacillus Reuteri for Treatment of Inflammatory Bowel Disease. Am. J. Transl. Res..

[143] Mukhopadhya I., Hansen R., El-Omar E.M., Hold G.L. (2012). IBD—What Role Do Proteobacteria Play?. Nat. Rev. Gastroenterol. Hepatol..

[144] Heidarian F., Noormohammadi Z., Aghdaei H.A., Alebouyeh M. (2017). Relative Abundance of Streptococcus Spp. and Its Association with Disease Activity in Inflammatory Bowel Disease Patients Compared with Controls. Arch. Clin. Infect. Dis..

[145] Rowan F., Docherty N.G., Murphy M., Murphy B., Calvin Coffey J., O’Connell P.R. (2010). Desulfovibrio Bacterial Species Are Increased in Ulcerative Colitis. Dis. Colon Rectum.

[146] Monk J.M., Lepp D., Zhang C.P., Wu W., Zarepoor L., Lu J.T., Pauls K.P., Tsao R., Wood G.A., Robinson L.E. (2016). Diets Enriched with Cranberry Beans Alter the Microbiota and Mitigate Colitis Severity and Associated Inflammation. J. Nutr. Biochem..

[147] Long X., Wong C.C., Tong L., Chu E.S.H., Ho Szeto C., Go M.Y.Y., Coker O.O., Chan A.W.H., Chan F.K.L., Sung J.J.Y. (2019). Peptostreptococcus Anaerobius Promotes Colorectal Carcinogenesis and Modulates Tumour Immunity. Nat. Microbiol..

[148] Vacca M., Celano G., Calabrese F.M., Portincasa P., Gobbetti M., De Angelis M. (2020). The Controversial Role of Human Gut Lachnospiraceae. Microorganisms.

[149] Li S., Wang Z., Yang Y., Yang S., Yao C., Liu K., Cui S., Zou Q., Sun H., Guo G. (2017). Lachnospiraceae Shift in the Microbial Community of Mice Faecal Sample Effects on Water Immersion Restraint Stress. AMB Express.

[150] Gieryńska M., Szulc-Dąbrowska L., Struzik J., Mielcarska M.B., Gregorczyk-Zboroch K.P. (2022). Integrity of the Intestinal Barrier: The Involvement of Epithelial Cells and Microbiota—A Mutual Relationship. Animals.

[151] Ihara S., Hirata Y., Serizawa T., Suzuki N., Sakitani K., Kinoshita H., Hayakawa Y., Nakagawa H., Ijichi H., Tateishi K. (2016). TGF-β Signaling in Dendritic Cells Governs Colonic Homeostasis by Controlling Epithelial Differentiation and the Luminal Microbiota. J. Immunol..

[152] Chamanara M., Rashidian A., Mehr S.E., Dehpour A.-R., Shirkohi R., Akbarian R., Abdollahi A., Rezayat S.-M. (2019). Melatonin Ameliorates TNBS-Induced Colitis in Rats through the Melatonin Receptors: Involvement of TLR4/MyD88/NF-ΚB Signalling Pathway. Inflammopharmacology.

[153] Tam J.S.Y., Coller J.K., Prestidge C.A., Bowen J.M. (2022). Investigation of TLR4 Antagonists for Prevention of Intestinal Inflammation. Inflammation.

[154] Niu S., Jing M., Wen J., Wei S., Li H., Li X., Ma X., Zhao Y. (2022). Jatrorrhizine Alleviates DSS-Induced Ulcerative Colitis by Regulating the Intestinal Barrier Function and Inhibiting TLR4/MyD88/NF-ΚB Signaling Pathway. Evid. Based Complement Altern. Med..

[155] Jia Z., Wang P., Xu Y., Feng G., Wang Q., He X., Song Y., Liu P., Chen J. (2022). Trypsin Inhibitor LH011 Inhibited DSS-Induced Mice Colitis via Alleviating Inflammation and Oxidative Stress. Front. Pharmacol..

[156] Akcan A., Kucuk C., Sozuer E., Esel D., Akyildiz H., Akgun H., Muhtaroglu S., Aritas Y. (2008). Melatonin Reduces Bacterial Translocation and Apoptosis in Trinitrobenzene Sulphonic Acid-Induced Colitis of Rats. World J. Gastroenterol..

[157] Chelakkot C., Ghim J., Ryu S.H. (2018). Mechanisms Regulating Intestinal Barrier Integrity and Its Pathological Implications. Exp. Mol. Med..

[158] Gardiner K.R., Halliday M.I., Barclay G.R., Milne L., Brown D., Stephens S., Maxwell R.J., Rowlands B.J. (1995). Significance of Systemic Endotoxaemia in Inflammatory Bowel Disease. Gut.

[159] Chojnacki C., Wisniewska-Jarosinska M., Walecka-Kapica E., Klupinska G., Jaworek J., Chojnacki J. (2011). Evaluation of Melatonin Effectiveness in the Adjuvant Treatment of Ulcerative Colitis. J. Physiol. Pharm..

[160] Shahrokh S., Qobadighadikolaei R., Abbasinazari M., Haghazali M., Aghdaei H.A., Abdi S., Balaii H., Khanzadeh-Moghaddam N., Zali M.R. (2021). Efficacy and Safety of Melatonin as an Adjunctive Therapy on Clinical, Biochemical, and Quality of Life in Patients with Ulcerative Colitis. Iran. J. Pharm. Res..

[161] Rakhimova O.Y., Rakhimova O.Y. (2010). Use of melatonin in combined treatment for inflammatory bowel diseases. Ter. Arkhiv.

[162] Chojnacki C., Błasiak J., Fichna J., Chojnacki J., Popławski T. (2018). Evaluation of Melatonin Secretion and Metabolism Exponents in Patients with Ulcerative and Lymphocytic Colitis. Molecules.

[163] Chojnacki C., Wiśniewska-Jarosińska M., Kulig G., Majsterek I., Reiter R.J., Chojnacki J. (2013). Evaluation of Enterochromaffin Cells and Melatonin Secretion Exponents in Ulcerative Colitis. World J. Gastroenterol..

[164] Knutson K.L., Spiegel K., Penev P., Van Cauter E. (2007). The Metabolic Consequences of Sleep Deprivation. Sleep Med. Rev..

[165] Nagai M., Hoshide S., Kario K. (2010). Sleep Duration as a Risk Factor for Cardiovascular Disease- a Review of the Recent Literature. Curr. Cardiol. Rev..

[166] Bishir M., Bhat A., Essa M.M., Ekpo O., Ihunwo A.O., Veeraraghavan V.P., Mohan S.K., Mahalakshmi A.M., Ray B., Tuladhar S. (2020). Sleep Deprivation and Neurological Disorders. Biomed Res. Int..

[167] Ibarra-Coronado E.G., Pantaleón-Martínez A.M., Velazquéz-Moctezuma J., Prospéro-García O., Méndez-Díaz M., Pérez-Tapia M., Pavón L., Morales-Montor J. (2015). The Bidirectional Relationship between Sleep and Immunity against Infections. J. Immunol. Res..

[168] Devaraj S., Hemarajata P., Versalovic J. (2013). The Human Gut Microbiome and Body Metabolism: Implications for Obesity and Diabetes. Clin. Chem..

[169] Jie Z., Xia H., Zhong S.-L., Feng Q., Li S., Liang S., Zhong H., Liu Z., Gao Y., Zhao H. (2017). The Gut Microbiome in Atherosclerotic Cardiovascular Disease. Nat. Commun..

[170] Tremlett H., Bauer K.C., Appel-Cresswell S., Finlay B.B., Waubant E. (2017). The Gut Microbiome in Human Neurological Disease: A Review. Ann. Neurol..

[171] Benedict C., Vogel H., Jonas W., Woting A., Blaut M., Schürmann A., Cedernaes J. (2016). Gut Microbiota and Glucometabolic Alterations in Response to Recurrent Partial Sleep Deprivation in Normal-Weight Young Individuals. Mol. Metab..

[172] Zhang S.L., Bai L., Goel N., Bailey A., Jang C.J., Bushman F.D., Meerlo P., Dinges D.F., Sehgal A. (2017). Human and Rat Gut Microbiome Composition Is Maintained Following Sleep Restriction. Proc. Natl. Acad. Sci. USA.

[173] Poroyko V.A., Carreras A., Khalyfa A., Khalyfa A.A., Leone V., Peris E., Almendros I., Gileles-Hillel A., Qiao Z., Hubert N. (2016). Chronic Sleep Disruption Alters Gut Microbiota, Induces Systemic and Adipose Tissue Inflammation and Insulin Resistance in Mice. Sci. Rep..

[174] Wang F., Zou J., Xu H., Huang W., Zhang X., Wei Z., Li X., Liu Y., Zou J., Liu F. (2022). Effects of Chronic Intermittent Hypoxia and Chronic Sleep Fragmentation on Gut Microbiome, Serum Metabolome, Liver and Adipose Tissue Morphology. Front. Endocrinol..

[175] Wang T., Wang Z., Cao J., Dong Y., Chen Y. (2022). Melatonin Prevents the Dysbiosis of Intestinal Microbiota in Sleep-Restricted Mice by Improving Oxidative Stress and Inhibiting Inflammation. Saudi J. Gastroenterol..

[176] Mezzatesta M.L., Gona F., Stefani S. (2012). Enterobacter Cloacae Complex: Clinical Impact and Emerging Antibiotic Resistance. Future Microbiol..

[177] Zhou Q., Zhang Y., Wang X., Yang R., Zhu X., Zhang Y., Chen C., Yuan H., Yang Z., Sun L. (2020). Gut Bacteria Akkermansia Is Associated with Reduced Risk of Obesity: Evidence from the American Gut Project. Nutr. Metab..

[178] Salín-Pascual R.J., Ortega-Soto H., Huerto-Delgadillo L., Camacho-Arroyo I., Roldán-Roldán G., Tamarkin L. (1988). The Effect of Total Sleep Deprivation on Plasma Melatonin and Cortisol in Healthy Human Volunteers. Sleep.

[179] Redwine L., Hauger R.L., Gillin J.C., Irwin M. (2000). Effects of Sleep and Sleep Deprivation on Interleukin-6, Growth Hormone, Cortisol, and Melatonin Levels in Humans1. J. Clin. Endocrinol. Metab..

[180] Honma A., Revell V.L., Gunn P.J., Davies S.K., Middleton B., Raynaud F.I., Skene D.J. (2020). Effect of Acute Total Sleep Deprivation on Plasma Melatonin, Cortisol and Metabolite Rhythms in Females. Eur. J. Neurosci..

[181] Jandhyala S.M., Talukdar R., Subramanyam C., Vuyyuru H., Sasikala M., Reddy D.N. (2015). Role of the Normal Gut Microbiota. World J. Gastroenterol..

[182] Gao T., Wang Z., Cao J., Dong Y., Chen Y. (2021). Melatonin Ameliorates Corticosterone-Mediated Oxidative Stress-Induced Colitis in Sleep-Deprived Mice Involving Gut Microbiota. Oxid. Med. Cell. Longev..

[183] Zhong X., Chen B., Yang L., Yang Z. (2018). Molecular and Physiological Roles of the Adaptor Protein CARD9 in Immunity. Cell. Death Dis..

[184] Park Y.-S., Chung S.-H., Lee S.-K., Kim J.-H., Kim J.-B., Kim T.-K., Kim D.-S., Baik H.-W. (2015). Melatonin Improves Experimental Colitis with Sleep Deprivation. Int. J. Mol. Med..

[185] Gao T., Wang Z., Cao J., Dong Y., Chen Y. (2022). The Role of Aeromonas-Goblet Cell Interactions in Melatonin-Mediated Improvements in Sleep Deprivation-Induced Colitis. Oxid. Med. Cell. Longev..

[186] Ananthakrishnan A.N., Long M.D., Martin C.F., Sandler R.S., Kappelman M.D. (2013). Sleep Disturbance and Risk of Active Disease in Patients with Crohn’s Disease and Ulcerative Colitis. Clin. Gastroenterol. Hepatol..

[187] Ranjbaran Z., Keefer L., Farhadi A., Stepanski E., Sedghi S., Keshavarzian A. (2007). Impact of Sleep Disturbances in Inflammatory Bowel Disease. J. Gastroenterol. Hepatol..

[188] Tang Y., Preuss F., Turek F.W., Jakate S., Keshavarzian A. (2009). Sleep Deprivation Worsens Inflammation and Delays Recovery in a Mouse Model of Colitis. Sleep Med..

[189] Brainard J., Gobel M., Scott B., Koeppen M., Eckle T. (2015). Health Implications of Disrupted Circadian Rhythms and the Potential for Daylight as Therapy. Anesthesiology.

[190] Walker W.H., Walton J.C., DeVries A.C., Nelson R.J. (2020). Circadian Rhythm Disruption and Mental Health. Transl. Psychiatry.

[191] Gaston K.J., Visser M.E., Hölker F. (2015). The Biological Impacts of Artificial Light at Night: The Research Challenge. Philos. Trans. R. Soc. B Biol. Sci..

[192] Voiculescu S.E., Le Duc D., Roșca A.E., Zeca V., Chiţimuș D.M., Arsene A.L., Drăgoi C.M., Nicolae A.C., Zăgrean L., Schöneberg T. (2016). Behavioral and Molecular Effects of Prenatal Continuous Light Exposure in the Adult Rat. Brain Res..

[193] Voigt R.M., Forsyth C.B., Green S.J., Mutlu E., Engen P., Vitaterna M.H., Turek F.W., Keshavarzian A. (2014). Circadian Disorganization Alters Intestinal Microbiota. PLoS ONE.

[194] Voigt R.M., Summa K.C., Forsyth C.B., Green S.J., Engen P., Naqib A., Vitaterna M.H., Turek F.W., Keshavarzian A. (2016). The Circadian Clock Mutation Promotes Intestinal Dysbiosis. Alcohol. Clin. Exp. Res..

[195] Deaver J.A., Eum S.Y., Toborek M. (2018). Circadian Disruption Changes Gut Microbiome Taxa and Functional Gene Composition. Front. Microbiol..

[196] Chu W., Zhai J., Xu J., Li S., Li W., Chen Z.-J., Du Y. (2020). Continuous Light-Induced PCOS-Like Changes in Reproduction, Metabolism, and Gut Microbiota in Sprague-Dawley Rats. Front. Microbiol..

[197] Malik I., Batra T., Das S., Kumar V. (2020). Light at Night Affects Gut Microbial Community and Negatively Impacts Host Physiology in Diurnal Animals: Evidence from Captive Zebra Finches. Microbiol. Res..

[198] Alghamdi B.S. (2018). The Neuroprotective Role of Melatonin in Neurological Disorders. J. Neurosci. Res..

[199] Zhang B., Chen T., Cao M., Yuan C., Reiter R.J., Zhao Z., Zhao Y., Chen L., Fan W., Wang X. (2022). Gut Microbiota Dysbiosis Induced by Decreasing Endogenous Melatonin Mediates the Pathogenesis of Alzheimer’s Disease and Obesity. Front. Immunol..

[200] Jing Y., Yang D., Bai F., Zhang C., Qin C., Li D., Wang L., Yang M., Chen Z., Li J. (2019). Melatonin Treatment Alleviates Spinal Cord Injury-Induced Gut Dysbiosis in Mice. J. Neurotrauma.

[201] Chooi Y.C., Ding C., Magkos F. (2019). The Epidemiology of Obesity. Metabolism.

[202] Duan M., Wang Y., Zhang Q., Zou R., Guo M., Zheng H. (2021). Characteristics of Gut Microbiota in People with Obesity. PLoS ONE.

[203] Bäckhed F., Manchester J.K., Semenkovich C.F., Gordon J.I. (2007). Mechanisms Underlying the Resistance to Diet-Induced Obesity in Germ-Free Mice. Proc. Natl. Acad. Sci. USA.

[204] Yin J., Li Y., Han H., Chen S., Gao J., Liu G., Wu X., Deng J., Yu Q., Huang X. (2018). Melatonin Reprogramming of Gut Microbiota Improves Lipid Dysmetabolism in High-Fat Diet-Fed Mice. J. Pineal Res..

[205] Xu P., Wang J., Hong F., Wang S., Jin X., Xue T., Jia L., Zhai Y. (2017). Melatonin Prevents Obesity through Modulation of Gut Microbiota in Mice. J. Pineal Res..

[206] Wang M.-K., Yue H.-Y., Cai J., Zhai Y.-J., Peng J.-H., Hui J.-F., Hou D.-Y., Li W.-P., Yang J.-S. (2021). COVID-19 and the Digestive System: A Comprehensive Review. World J. Clin. Cases.

[207] Delavari A., Asgari S., Alimohamadi Y., Vosoogh-Moghaddam A., Sadeghi A., Shahrousvand S., Zakeri A., Moradzadeh R., Akbarpour S. (2022). XsGastrointestinal Symptoms Are Associated with a Lower Risk of Hospitalization and Mortality and Outcomes in COVID-19. BMC Gastroenterol..

[208] van der Lelie D., Taghavi S. (2020). COVID-19 and the Gut Microbiome: More than a Gut Feeling. mSystems.

[209] Zuo T., Zhang F., Lui G.C.Y., Yeoh Y.K., Li A.Y.L., Zhan H., Wan Y., Chung A.C.K., Cheung C.P., Chen N. (2020). Alterations in Gut Microbiota of Patients With COVID-19 During Time of Hospitalization. Gastroenterology.

[210] Zuo T., Wu X., Wen W., Lan P. (2021). Gut Microbiome Alterations in COVID-19. Genom. Proteom. Bioinform..

[211] Wang B., Zhang L., Wang Y., Dai T., Qin Z., Zhou F., Zhang L. (2022). Alterations in Microbiota of Patients with COVID-19: Potential Mechanisms and Therapeutic Interventions. Signal Transduct. Target..

[212] Yeoh Y.K., Zuo T., Lui G.C.-Y., Zhang F., Liu Q., Li A.Y., Chung A.C., Cheung C.P., Tso E.Y., Fung K.S. (2021). Gut Microbiota Composition Reflects Disease Severity and Dysfunctional Immune Responses in Patients with COVID-19. Gut.

[213] Stawinski P., Dziadkowiec K.N., Marcus A. (2021). COVID-19-Induced Colitis: A Novel Relationship During Troubling Times. Cureus.

[214] Rutigliani M., Bozzo M., Barberis A., Greppi M., Anelli E., Castellaro L., Bonsignore A., Azzinnaro A., Pesce S., Filauro M. (2022). Case Report: A Peculiar Case of Inflammatory Colitis After SARS-CoV-2 Infection. Front. Immunol..

[215] Carvalho A., Alqusairi R., Adams A., Paul M., Kothari N., Peters S., DeBenedet A.T. (2020). SARS-CoV-2 Gastrointestinal Infection Causing Hemorrhagic Colitis: Implications for Detection and Transmission of COVID-19 Disease. Am. J. Gastroenterol..

[216] Zhang R., Wang X., Ni L., Di X., Ma B., Niu S., Liu C., Reiter R.J. (2020). COVID-19: Melatonin as a Potential Adjuvant Treatment. Life Sci.

[217] Reiter R.J., Abreu-Gonzalez P., Marik P.E., Dominguez-Rodriguez A. (2020). Therapeutic Algorithm for Use of Melatonin in Patients With COVID-19. Front. Med..

[218] Yayıcı Köken Ö., Gültutan P., Güngören M.S., Bayhan G.İ., Yılmaz D., Gürkaş E., Özyürek H., Çıtak Kurt A.N. (2021). Impact of COVID-19 on Serum Melatonin Levels and Sleep Parameters in Children. Turk. J. Med. Sci..

[219] Camp O.G., Bai D., Gonullu D.C., Nayak N., Abu-Soud H.M. (2021). Melatonin Interferes with COVID-19 at Several Distinct ROS-Related Steps. J. Inorg. Biochem..

[220] Farnoosh G., Akbariqomi M., Badri T., Bagheri M., Izadi M., Saeedi-Boroujeni A., Rezaie E., Ghaleh H.E.G., Aghamollaei H., Fasihi-Ramandi M. (2022). Efficacy of a Low Dose of Melatonin as an Adjunctive Therapy in Hospitalized Patients with COVID-19: A Randomized, Double-Blind Clinical Trial. Arch. Med. Res..

[221] Hasan Z.T., Atrakji D.M.Q.Y.M.A.A., Mehuaiden D.A.K. (2022). The Effect of Melatonin on Thrombosis, Sepsis and Mortality Rate in COVID-19 Patients. Int. J. Infect. Dis..

[222] Mousavi S.A., Heydari K., Mehravaran H., Saeedi M., Alizadeh-Navaei R., Hedayatizadeh-Omran A., Shamshirian A. (2022). Melatonin Effects on Sleep Quality and Outcomes of COVID-19 Patients: An Open-Label, Randomized, Controlled Trial. J. Med. Virol..

